# Efficacy and acceptability of psilocybin for primary or secondary depression: A systematic review and meta-analysis of randomized controlled trials

**DOI:** 10.3389/fpsyt.2024.1359088

**Published:** 2024-02-15

**Authors:** Shuping Fang, Xin Yang, Wei Zhang

**Affiliations:** ^1^ Mental Health Center of West China Hospital, Sichuan University, Chengdu, China; ^2^ West China Biomedical Big Data Center, West China Hospital, Sichuan University, Chengdu, China; ^3^ Med-X Center for Informatics, Sichuan University, Chengdu, China

**Keywords:** psilocybin, depression, efficacy, acceptability, systematic review

## Abstract

**Introduction:**

Psilocybin is a classic psychedelics, which has been shown to have antidepressant effects by many studies in recent years. In this study, we aim to evaluate the efficacy, acceptability and tolerability of psilocybin in the treatment of primary (major depressive disorder) or secondary (experiencing distress related to life-threatening diagnoses and terminal illness) depression.

**Methods:**

We searched PubMed, EMBASE, Web of Science, Cochrane Library and ClinicalTrials.gov for clinical trials of psilocybin for depression (updated to 4 October, 2023). Effect size Hedges’ g was used as an indicator of efficacy, and other outcomes included response rate, drop-out rate, and adverse events.

**Results:**

A total of 10 studies were finally included in systematic review. 8 studies were included in the meta-analysis, involving a total of 524 adult patients, and produced a large effect size in favor of psilocybin (Hedge’s g =-0.89, 95% CI -1.25~-0.53, I² = 70.19%, P<0.01). The therapeutic effects of psilocybin increase with increasing doses. Adverse events caused by psilocybin are generally transient and reversible, but serious adverse events also may occur.

**Discussion:**

Our study shows that psilocybin has both short-term and long-term antidepressant effects and holds promise as a potential complementary or alternative therapy for depression, probably. Further research may reveal more about its therapeutic potential.

## 1 Introduction

Depression, a prevalent mood disorder affecting approximately 5% of adults globally (1), is characterized by diminished mood or loss of well-being, accompanied by cognitive and behavioral manifestations (2). In 2008, the World Health Organization ranked depression as the third-largest contributor to the global disease burden and projected its potential to become the leading cause by 2030 (3). Each year, an estimated 12 billion workdays are lost due to depression and anxiety, resulting in a global economic cost of approximately $1 trillion annually (4). This substantial impact on the family and society has far-reaching consequences.

Effective interventions for depression encompass psychotherapy, pharmacotherapy, electroconvulsive therapy, and comprehensive approaches (5, 6). In comparison to psychotherapy, pharmacological treatment offers a more expeditious and accessible means of alleviating depressive symptoms (7, 8). Currently, selective serotonin reuptake inhibitors (SSRIs) and serotonin-norepinephrine reuptake inhibitors (SNRIs) are extensively employed as first-line antidepressants, typically requiring a minimum of two weeks to demonstrate efficacy, with a response rate of approximately 47% and a remission rate as low as 36.8% (9, 10). In contrast, electroconvulsive therapy (ECT) exhibits a more rapid onset of action and greater efficacy with definitive outcomes; however, it also presents challenges such as intricate implementation procedures, post-treatment cognitive impairment, and the presence of treatment-related stigma (11, 12). Currently, treatment-resistant depression (TRD) affects approximately 30 to 40% of patients diagnosed with depression (9, 10). Therefore, it is crucial to investigate novel treatment options that exhibit rapid onset, pronounced efficacy and tolerable side effects. In recent years, the antidepressant properties of psilocybin, a classic psychedelic drug, have rekindled the interest of researchers (13–16) and garnered approval from the U.S. Food and Drug Administration (FDA) as a “breakthrough therapy” for treating depression, including treatment-resistant depression (17).

The completion of numerous clinical trials has been observed in the investigation of the antidepressant properties exhibited by psilocybin (18–27). Many meta-analyses have consistently demonstrated that psilocybin exerts a positive impact on depression (28–33). However, acceptability was not reported in detail in these meta-analyses. In light of recent completion of new clinical trials, our meta-analysis aims to evaluate both the efficacy and acceptability of psilocybin for primary (major depressive disorder) or secondary (experiencing distress related to life-threatening diagnoses and terminal illness) depression.

## 2 Methods

The study protocol has been registered with PROSPERO (CRD 42023369397) and adheres to the Cochrane Handbook for Systematic Reviews of Interventions and PRISMA 2020 statement for systematic review reporting (34, 35).

### 2.1 Search strategy

We searched Embase, PubMed, Web of Science, Cochrane Library and ClinicalTrials.gov from inception up until October 06, 2023. Searches were performed without year of publication restriction, but the language was restricted to English. We used the following search strategy: [(depression [MeSH Terms]) OR (depressive disorder [MeSH Terms]) OR (depress*) OR (mood disorder*) OR (affective disorder) OR (unipolar depression)] AND [(psilocybin [MeSH Terms]) OR (psilocybin) OR (psilocybine) OR (psiloc*)] AND [(Randomized Controlled Trial[Publication Type]) OR (Randomized Controlled Trial) OR (Controlled Clinical Trials, Randomized) OR (Random Allocation) OR (Double-blind) OR (Single-blind) OR (Placebo)].

### 2.2 Selection procedure

To eliminate duplicate articles, all records were imported into the EndNote reference management software and identified duplicates were subsequently removed. The remaining articles were independently screened by two authors (SF and XY) based on relevance of title, abstract, article type, and full-text. Any discrepancies in the selection process were resolved through consensus between the two authors. In cases where a consensus could not be reached, a third researcher was consulted.

The PICOS (Participant, Prevention, Comparison, Outcomes and Study Design) rules mentioned in PRISMA are the basis for the inclusion and exclusion criteria below.

Inclusion criteria:

(a) Participant: Male or female patients aged 18 years or older, diagnosed with major depressive disorders and/or experiencing distress related to life-threatening diagnoses and terminal illness. Diagnoses were based on versions of the DSM or the SCID.
(b) Intervention: Psilocybin.
(c) Comparison: Placebo control or low-dose psilocybin control.
(d) Outcome: The primary objective of this study was to evaluate the efficacy, acceptability, and tolerability of psilocybin in ameliorating depression and depressive symptoms. The efficacy of ameliorating depression and depressive symptoms is evaluated through the utilization of effective psychological assessment tools, encompassing: Montgomery–Asberg Depression Rating Scale (MADRS) (36), Hamilton Depression Scale (HAMD) (37), Beck Depression Scale (BDI) (38), Quick Inventory of Depressive Symptomatology (QIDS) (39), the clinician-administered GRID-HAM-D-17 (40),and these assessment tools serve as widely adopted indicators of the severity of depression. To assess the acceptability and tolerability of psilocybin, we conducted an analysis encompassing both the incidence of adverse events and the rate of discontinuation for any cause (41).
(e) Study design: Only randomized controlled trials (RCTs) were included, non-randomized studies, open-label studies, animal studies, reviews, meta-analyses and systematic reviews were excluded.

Participants under the age of 18 were included, while non-randomized controlled trials and articles failing to meet the aforementioned inclusion criteria were excluded.

### 2.3 Data extraction

Two authors independently reviewed and extracted relevant data, including literature information (title, first author, publication date, country or region), demographic and clinical characteristics of participants (age, sex, ethnicity, diagnostic criteria), methodological details (sample size, study type, psilocybin dose, scales used, main assessment points, trial duration), and adverse events. For the meta-analysis, we extracted mean ± standard deviation of depression scale scores (MADRS, HAMD, BDI, QIDS) before and after treatment. If the necessary data were not fully described in the publication, we made efforts to extract the data from the provided figure using Engauge Digitizer software (42, 43) or contacted the corresponding authors of the study. Relevant studies that lacked accessible data through these methods were excluded from our analysis.

### 2.4 Risk of bias assessment

To evaluate the risk of bias in the included RCTs, two authors independently used the revised Cochrane risk-of-bias tool for randomized trials (44) and Review Manager software (RevMan 5.4.1) to assess seven domains: (1) random sequence generation (selection bias), (2) allocation concealment (selection bias), (3) blinding of participants and personnel (performance bias), (4) blinding of outcome assessment (detection bias), (5) incomplete outcome data (attrition bias), (6) selective reporting (reporting bias), and (7) other sources of bias. The risk of bias for each domain was assessed as low, unclear, or high. Disagreements were resolved through negotiation between the two authors and, if unresolved, a third researcher was consulted. Subsequently, a meta-analysis was conducted using appropriate statistical methods to combine the findings from individual studies.

### 2.5 Statistical analysis

Meta-analyses were conducted using Stata/MP 17.0. The mean and standard deviation (M ± SD) of continuous variables for the experimental and control groups were extracted or indirectly derived from the included studies. The standardized mean difference was employed as the effect size for continuous variables evaluated using diverse scales. In order to mitigate the influence of a limited sample size on the conclusions, Hedges’s g was selected as the effect size (45). The weighted mean difference (WMD) was employed as the effect size for data obtained through identical assessment methods, such as heart rate and blood pressure. Binary variables, including response rates, dropout rates, and adverse events, were analyzed using odds ratios (OR).

Heterogeneity refers to the differences between the included studies. I-squared (I²) (46) was used to evaluate the size of heterogeneity and was interpreted as the following thresholds: I² 0-40%: possibly not important; 30-60%: possibly indicating moderate heterogeneity; 50-90%: possibly indicating significant heterogeneity; 75-100%: considerable heterogeneity (34). Given the potential variability in intervention effects across studies, we applied a random-effects model for most of the meta-analyses conducted, while a fixed-effect model was used specifically for examining the effects of psilocybin on heart rate and blood pressure (47).

Subgroup analyses were conducted to investigate the sources of heterogeneity, including the disease type of the subjects, the range of drug dosage, and the duration of drug efficacy. Furthermore, a leave-one-out method was employed for sensitivity analysis to assess the robustness of the meta-analysis.

Funnel plots and Egger’s test (48) were used to evaluate publication bias.

## 3.Results

### 3.1 Search results

**Figure 1:**
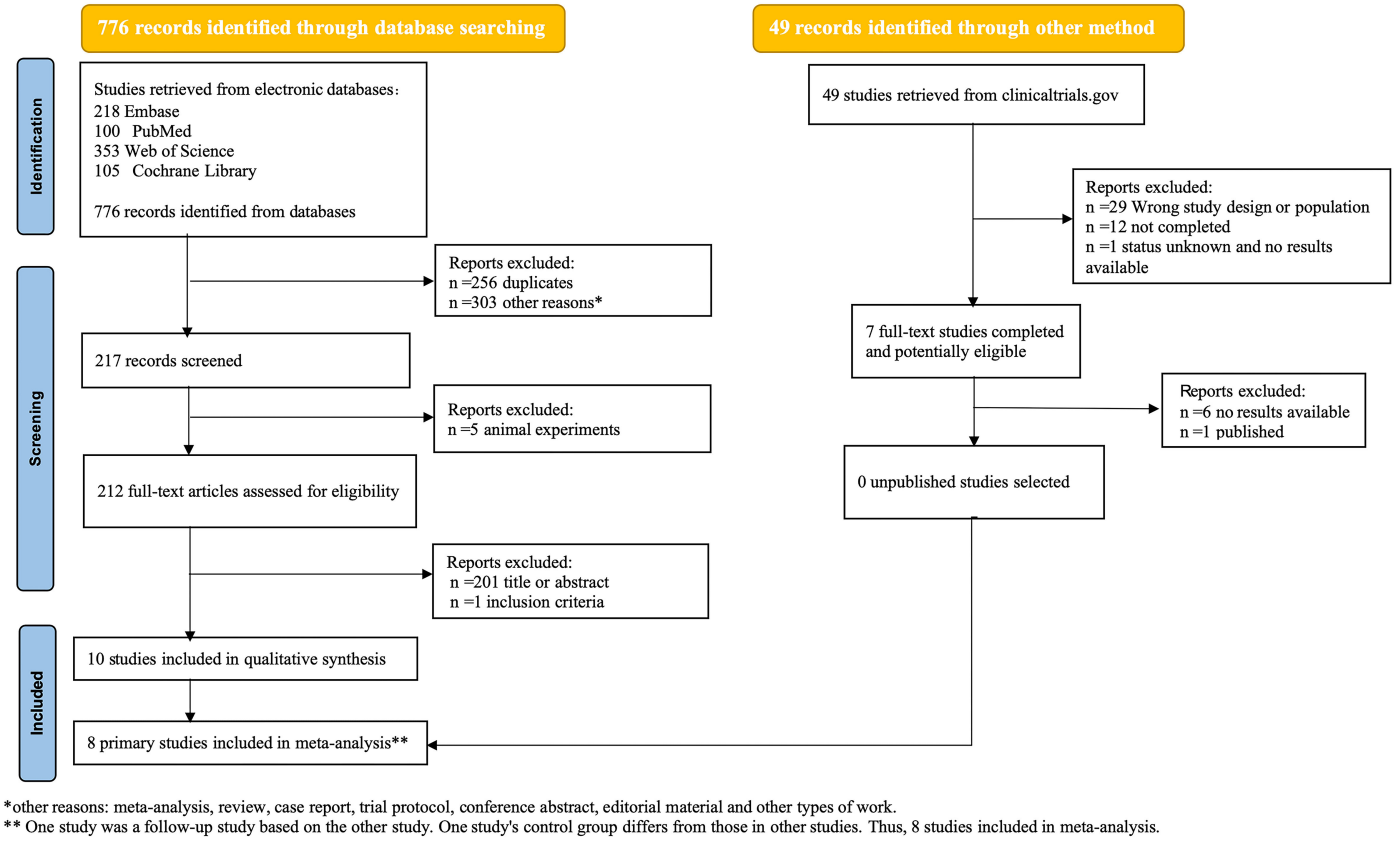
Prisma flow-chart of study selection process.

We retrieved 776 potentially relevant published articles from databases and 49 relevant clinical research trials from the website(clinicaltrials.gov). After undergoing independent screening by two investigators, a total of 10 studies were deemed eligible for inclusion in the systematic review, of which 8 studies were included in the meta-analysis. The reasons for exclusion have been elucidated in the PRISMA flow-chart (**Figure 1**).

### 3.2 Main characteristics of included studies

In our study, we included a total of 10 studies (18–27), with Gukasyan’s study (23) being a 12-month follow-up of Davis et al.’s research (22). Therefore, we considered these two studies (22, 23) as two stages of the same research. It should be noted that Carhart-Harris et al.’s study (21) used escitalopram as a control group, which is quite different from the control groups used in other studies. Considering that including the active drug(escitalopram) as a control group in the meta-analysis may affect the results of the meta-analysis and underestimate the efficacy of psilocybin, we excluded it from the meta-analysis. Ultimately, we included 8 randomized controlled trials (18–20, 22, 24–27) in our meta-analysis, which involved a total of 524 adult patients.

These studies exhibited variations in experimental design, with three studies being double-blind randomized controlled trials (24, 25, 27) and two studies being randomized, double-blind, cross-over design trials (19, 20), one study was a randomized double-blind within-subject crossover design experiment (18), one was a randomized waitlist controlled experiment (22), and finally one study employed a double-blind placebo-controlled within-subject fixed-order experiment (26). Notably, although participants in each study were instructed to gradually reduce and discontinue antidepressants before the baseline visit to mitigate the potential impact of antidepressants on trial outcomes, Von Rotz et al.’s study reported that 38 individuals had stopped antidepressant medication at baseline, 12 were using one antidepressant, and 2 were using more than one antidepressant. Baseline antidepressant medication usage by participants was not reported in other studies.

Furthermore, the subjects in these studies were diverse, with three of them (18–20) involving patients experiencing distress related to life-threatening diagnoses and terminal illness, categorized as secondary depression. The remaining five studies focused on subjects with primary depression, of which four studies (22, 25–27) involved patients diagnosed with major depressive disorder (MDD), while one study (24) included individuals suffering from treatment-resistant depression (TRD). It’s worth noting that in the study by Goodwin et al., TRD was defined as the failure of two courses of antidepressants from differing classes. Participants not only had to meet the criteria for TRD but also had a current episode of depression that had not responded to two to four adequate trials in terms of both dose and duration (≥8 weeks) of treatment according to the Massachusetts General Hospital Antidepressant Treatment Response Questionnaire.

Among these studies, seven studies (18–20, 24–27) employed a single treatment approach, whereas one study (22) administered two treatments separated by a one-week interval. **Table 1** summarizes the characteristics of the included studies.

**Table 1:** Characteristics of the included studies.

Study	Study type	Object	Sample size	Age(range)/(mean ± SD)	Gender (%female)	Race (%white)	Intervention/ Control	Scale	Point (week)
Grob et al. (18)	RCT within-subject crossover trial	Cancer+ Anx DSM-IV diagnosed Duration of illness: not reported	12	36to58	11 (92%)	NA	Psilocybin(0.2mg/kg)/ Niacin(250mg)	BDI	2
Griffiths et al. (19)	RCT crossover trial	Cancer + Dep (69%)/Anx (63%) DSM-IV diagnosed Duration of illness: not reported	51	56.3 ± 9.99	25 (49%)	48 (94%)	Psilocybin(22or30mg/70kg)/ Psilocybin(1or3mg/70kg)	HAM-D	5
Ross et al. (20)	RCT crossover trial	Cancer + Dep (28%)/ Anx (62%)/GAD (10%) DSM-IV, SCID- IV diagnosed Duration of illness: not reported	29	56.3 ± 12.93	18 (62%)	26 (90%)	Psilocybin(0.3mg/kg)/ Niacin(250mg)	BDI	2
Carhart-Harris et al. (21)	RCT	moderate-severe MDD confirmation by patient’s general physician (HAM-D-17 ≥17) Duration of illness: range 2-46 (year)	59	41.2 ± 10.9	20 (34%)	52 (88%)	Psilocybin(25mg)/ Escitalopram(10or20mg)	QIDS-SR-16	6
Davis et al. (22)	RCT Waiting-list	moderate or severe MDD DSM-5, SCID-5 diagnosed (GRID-HAMD ≥17) Duration of illness: 21.5 ± 12.2(year)	24	39.8 ± 12.2	16 (67%)	22 (92%)	Psilocybin(20or30mg/70kg)/ Psilocybin(20or30mg/70kg)^*^	HAM-D	1, 4
Goodwin et al. (24)	RCT	TRD (failure of two courses of antidepressants from differing classes) DSM-5 diagnosed Lifetime depressive episodes: 6.9 ± 7.6 Recurrent MDD episode: 222(95%) Duration of illness: not reported	233	39.8 ± 12.2	121 (52%)	215 (92%)	Psilocybin(25mg)/ Psilocybin(10mg)/ Psilocybin(1mg)	MADRS	3
Von Rotz et al. (27)	RCT	MDD MINI diagnosed (MADRS score: 10-40) Duration of illness: not reported	52	36.8 ± 10.3	33 (63%)	49 (94%)	Psilocybin (0.215 mg/kg)/ Pure mannitol (0.215 mg/kg)	MADRS BDI	2
Sloshower et al. (26)	Placebo-controlled, within-subject, fixed-order	MDD DSM-5, SCID-5 diagnosed Duration of illness: 20 ± 12(year)	19	42.8 ± 13.8	13 (68%)	16 (84%)	Psilocybin (0.3mg/kg)/ microcrystalline cellulose	GRID-HAM-D	2
Raison et al. (25)	RCT	MDD DSM-5, SCID-5 diagnosed (MADRS score≥28) Duration of current depressive episode: range 0.48-2.78(year) Duration of illness: not reported	104	41.1 ± 11.3	52 (50%)	93 (89.4%)	Psilocybin(25mg)/ Niacin(100mg)	MADRS	6

RCT, Randomized controlled trial; Dep, depressed mood; Anx, anxiety disorder; GAD, generalized anxiety disorder; MDD, major depressive disorder; TRD, treatment-resistant depression; BDI, Beck depression inventory; HAM-D, Hamilton depression rating scale; QIDS-SR-16, 16-item Quick Inventory of Depressive Symptomatology–Self-Report; MADRS, Montgomery-Asberg depression rating scale;

*In this trial, both psilocybin sessions (session 1: 20 mg/70 kg; session 2: 30 mg/70 kg) were applied to immediate treatment and delayed treatment.

### 3.3 Risk of bias assessment

**Figure 2:**
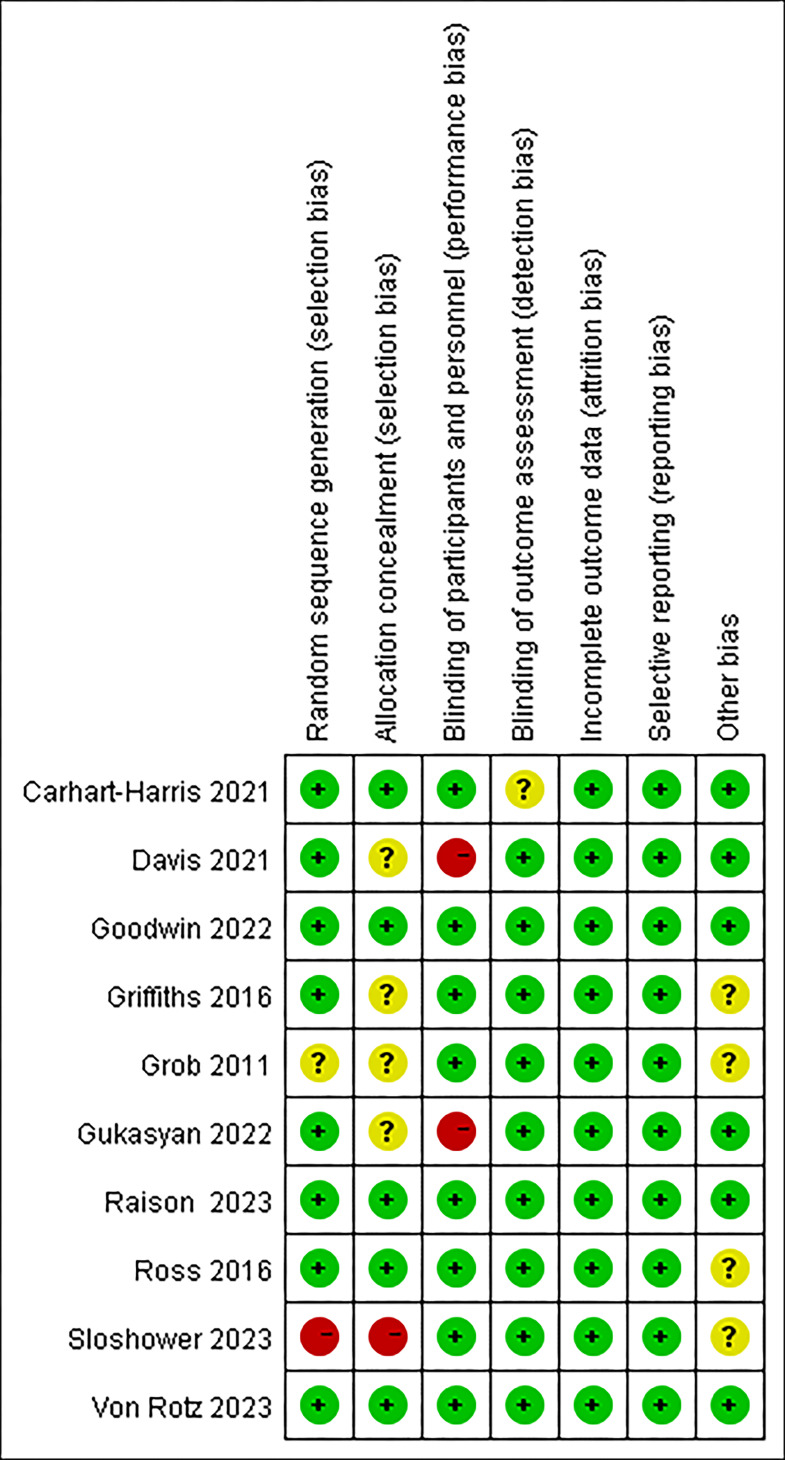
Risk of bias assessment of the included studies. Green indicates low risk of bias, yellow indicates unclear risk of bias, and red indicates high risk of bias.

The overview of the bias risk assessment results is presented in **Figure 2**. In evaluating the risk of bias, ten included studies were assessed. Three studies (22, 23, 26) were judged to have a high risk of bias, four studies (18–21) were judged to have an unclear risk of bias, and three studies were judged to have a low risk of bias. Regarding the generation of random sequence, eight (19–25, 27) were judged to have a low risk of bias due to the correct description of randomization methods, while one (18) did not report the sequence generation process. One study (26) used a fixed-sequence study design with placebo followed by psilocybin, which did not involve randomization and was therefore rated as high risk of bias. For allocation concealment, the risk of bias was low in five studies (20, 21, 24, 25, 27)and unknown in four (18, 19, 22, 23) and high in one (26). For blinding of participants and personnel, eight studies (18–20, 22, 24–27) had a low risk of bias, while two (22, 23) were considered high risk because they used a randomized waiting list control design, which allowed patients to know which treatment they were receiving. For blinding of outcome assessment, the risk of bias was low in nine studies (18–20, 22–27), while Carhart-Harris et al.’s study (21) did not specify whether blinding was performed. For incomplete outcome data and selective reporting, all studies were rated as having a low risk of bias. For other sources of bias, six studies (21–25, 27) were rated as having a low risk of bias, and fore studies (18–20, 26)were rated as unclear because they all used a crossover trial design.

### 3.4 Effect of psilocybin on primary or secondary depression

**Figure 3:**
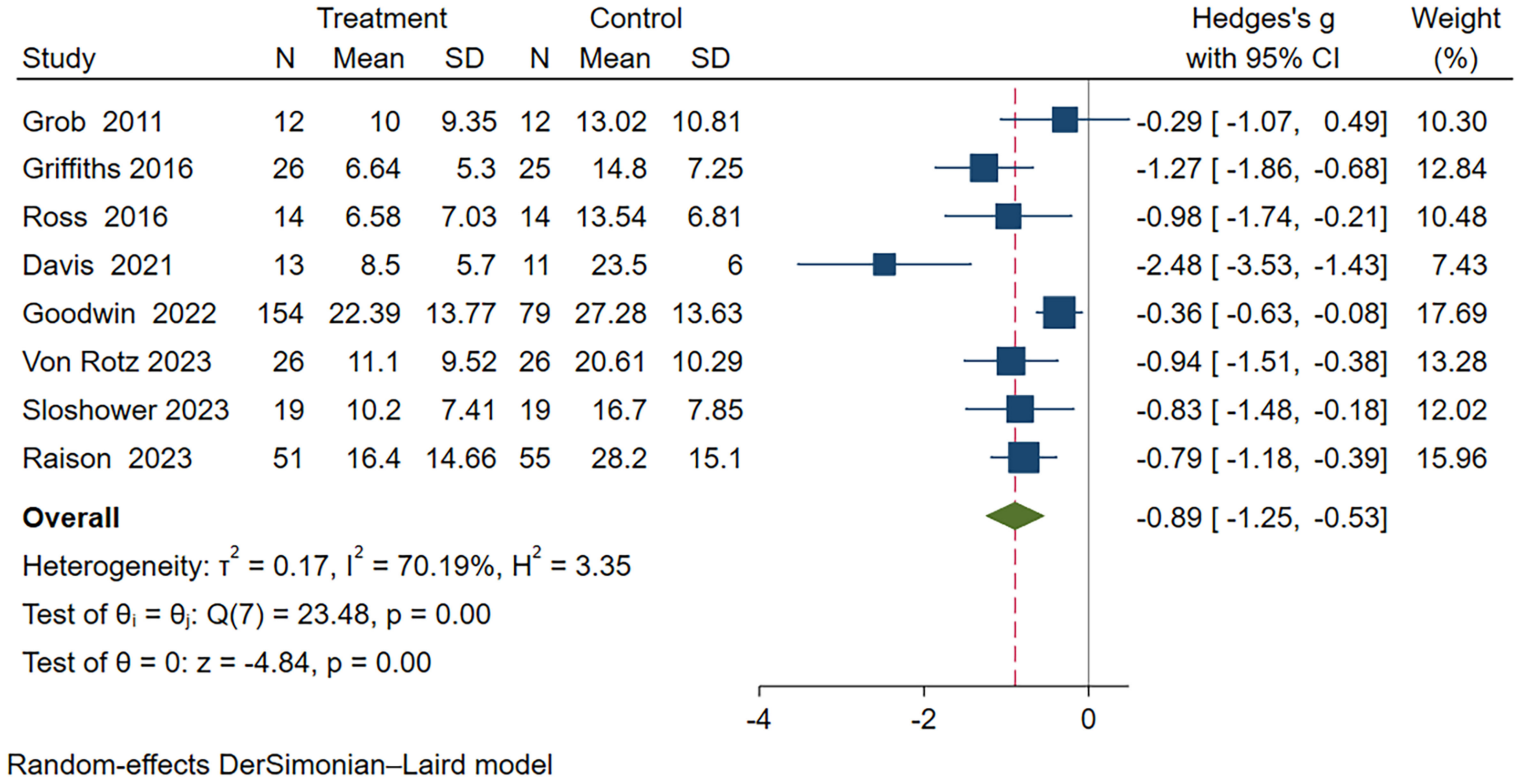
Meta-analysis of psilocybin compared to placebo/low-dose psilocybin for treating primary or secondary depression.

In this meta-analysis, a random-effects model was used to analyze the eight included studies (18–20, 22, 24–27), which demonstrated a significant therapeutic effect of psilocybin on depression (Hedge’s g = -0.89, 95% CI -1.25~-0.53, I² = 70.19%, P < 0.01), as detailed in the **Figure 3**. Heterogeneity analysis revealed significant heterogeneity between studies (I² = 70.19%). We attempted to conduct subgroup analyses to investigate the sources and factors that may have contributed to the observed heterogeneity between studies.

### 3.5 Subgroup analysis

**Figure 4:**
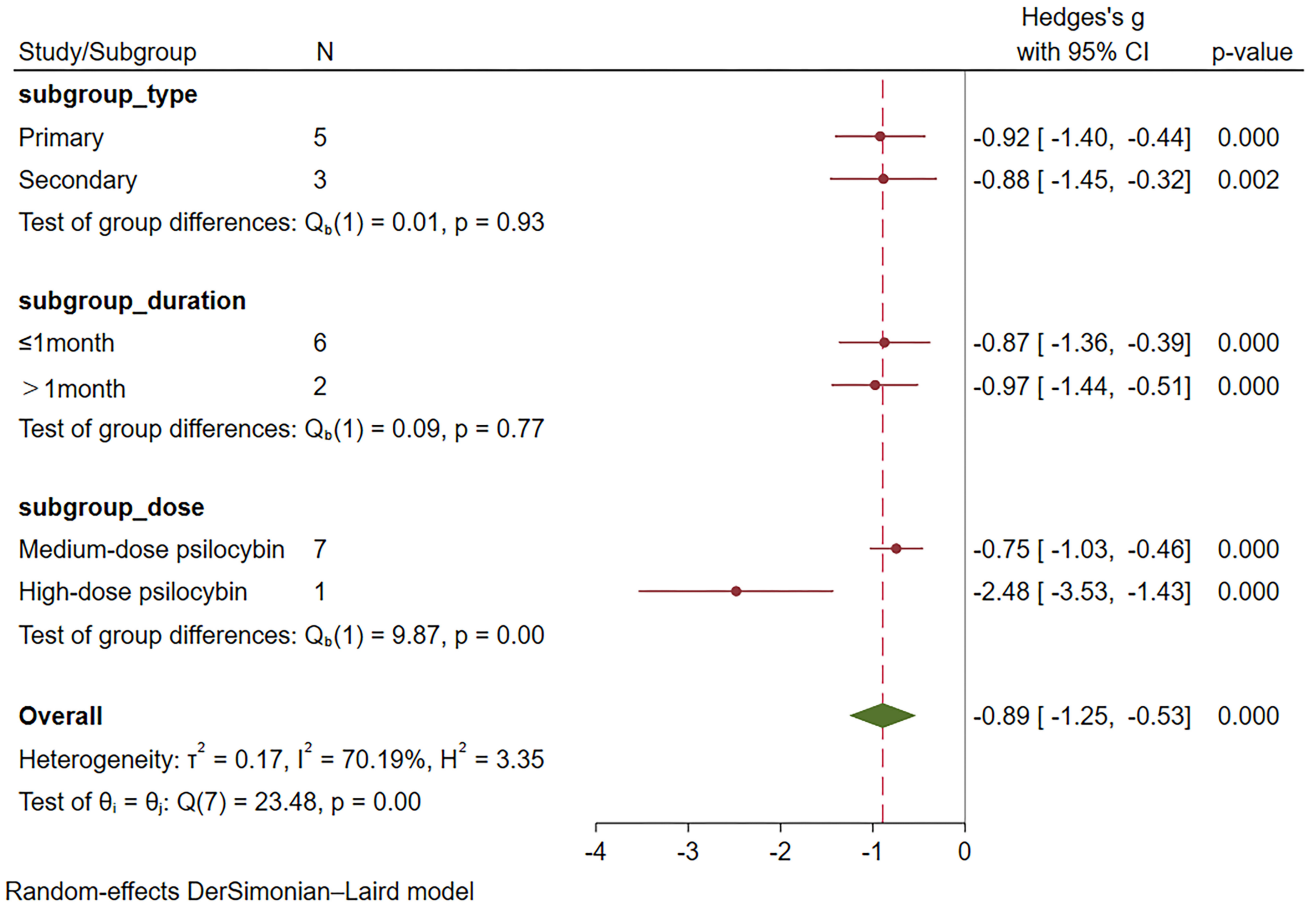
Overall subgroup analysis results based on participant type, dose and duration.

**Figure 5:**
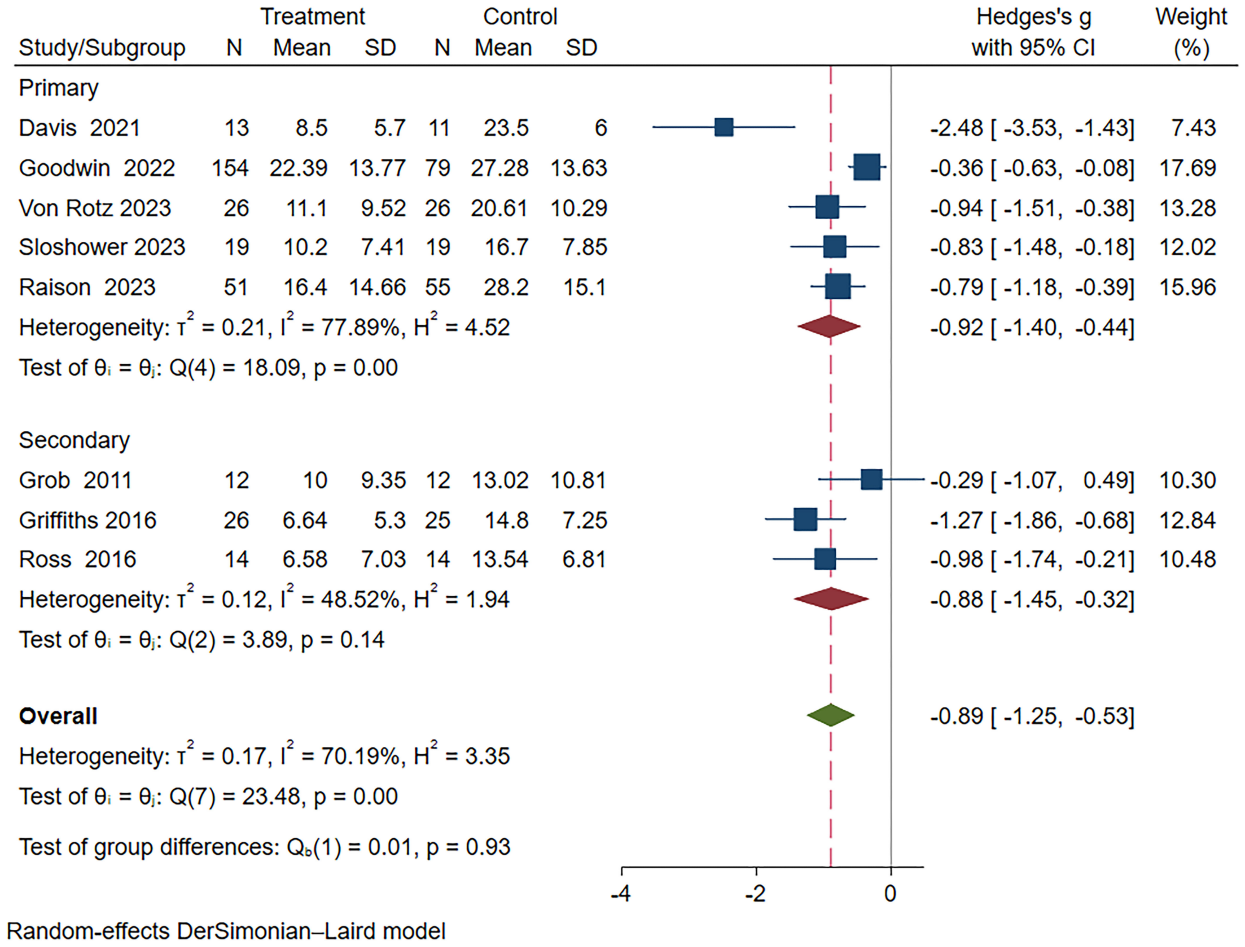
Meta-analysis of psilocybin compared to placebo/low-dose psilocybin for treating primary or secondary depression: subgroup analysis based on participant type.

Subgroup meta-analyses were performed based on distinct participant disease types, drug action timeframes, and drug dosages. **Table 2** provides comprehensive grouping details, and **Figure 4** provides the overall subgroup analysis results. The subgroup analysis was conducted based on different subject types, revealing that psilocybin exhibits a significant impact on primary depression (Hedges’ g= -0.92, 95% CI: -1.4 ~ -0.44, I² = 77.89%, p < 0.01). Notably, its efficacy surpasses that observed in cases of secondary depression (Hedges’ g= -0.88, 95% CI: -1.45~ -0.32, I² = 48.52%, p =0.14) (**Figure 5**).

**Table 2:** Details of subgroup meta-analysis based on participant disease type, drug dose, and duration of drug action.

Subgroup	Group details	Number of included studies	Number of psilocybin group	Number of control group	Effect size(95%CI)	P value	I²(%)
Disease type	Primary	5	263	190	-0.92(-1.4, -0.44)	<0.01	77.89
	Secondary	3	52	51	-0.88(-1.45, -0.32)	0.14	48.52
Dose	Low-dose psilocybin^*^	0	—	—	—	—	—
	Medium-dose psilocybin	7	302	230	-0.75(-1.03, -0.46)	0.06	50.12
	High-dose psilocybin	1	13	11	-2.48(-3.53, -1.43)	—	—
Duration	≤1Month	6	238	161	-0.87(-1.36, -0.39)	<0.01	73.69
	>1Month	2	77	80	-0.97(-1.44, -0.51)	0.18	43.27

*Low-dose psilocybin: ≤5mg; Medium-dose psilocybin: 10-30mg; High-dose psilocybin: >30mg.

**Figure 6:**
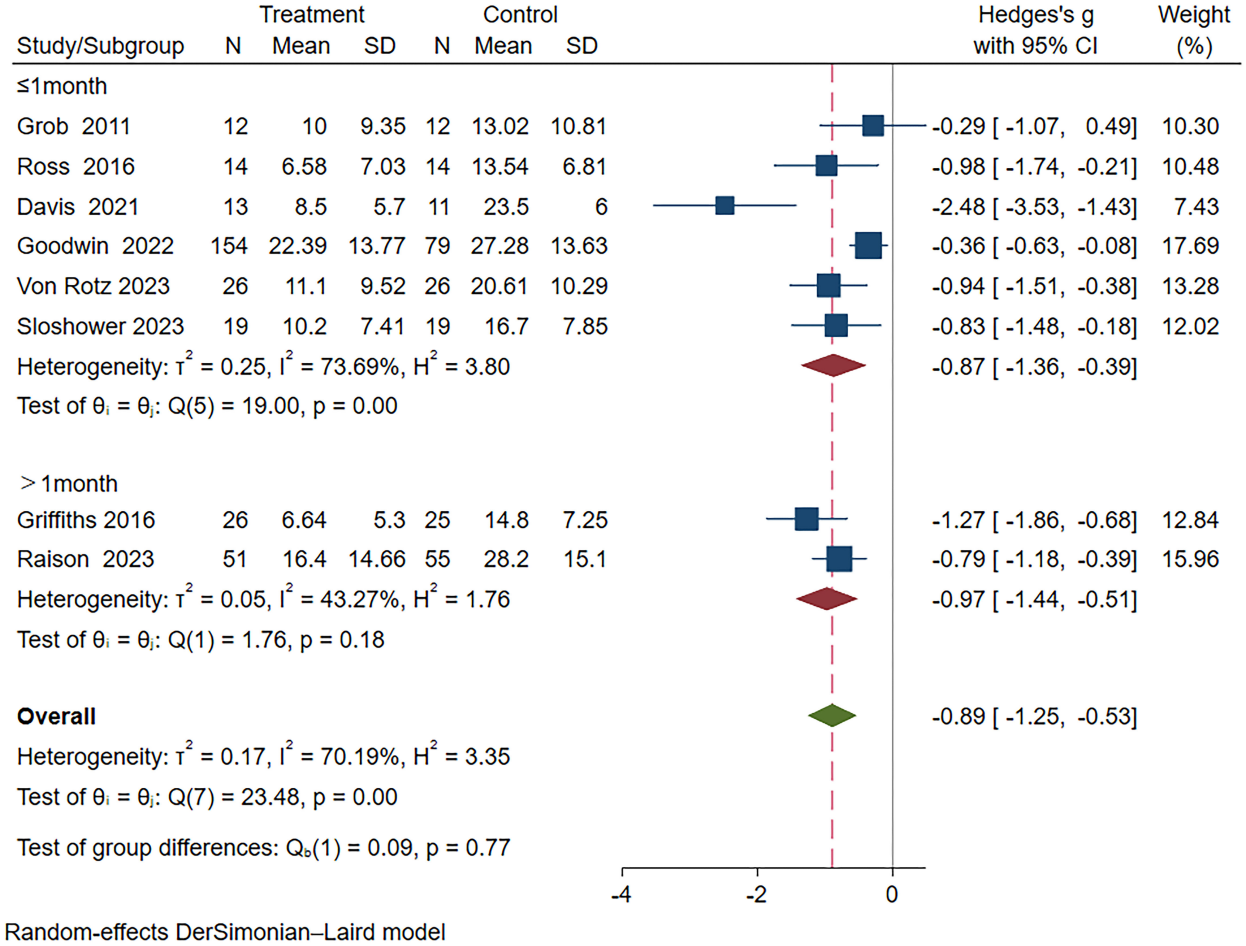
Meta-analysis of psilocybin compared to placebo/low-dose psilocybin for treating primary or secondary depression: subgroup analysis based on duration time.

Furthermore, the subgroup analysis based on duration revealed significant differences between both subgroups; however, the long-term subgroup (>1 month) (Hedges’ g =-0.97, 95% CI: -1.44 ~ -0.51, I²=43.27%, p=0.18) exhibited superior efficacy compared to the short-term subgroup (≤1 month) (Hedges’ g=-0.87, 95% CI: -1.36 ~ -0.39, I²=73.69%, p<0.01) (**Figure 6**).

**Figure 7:**
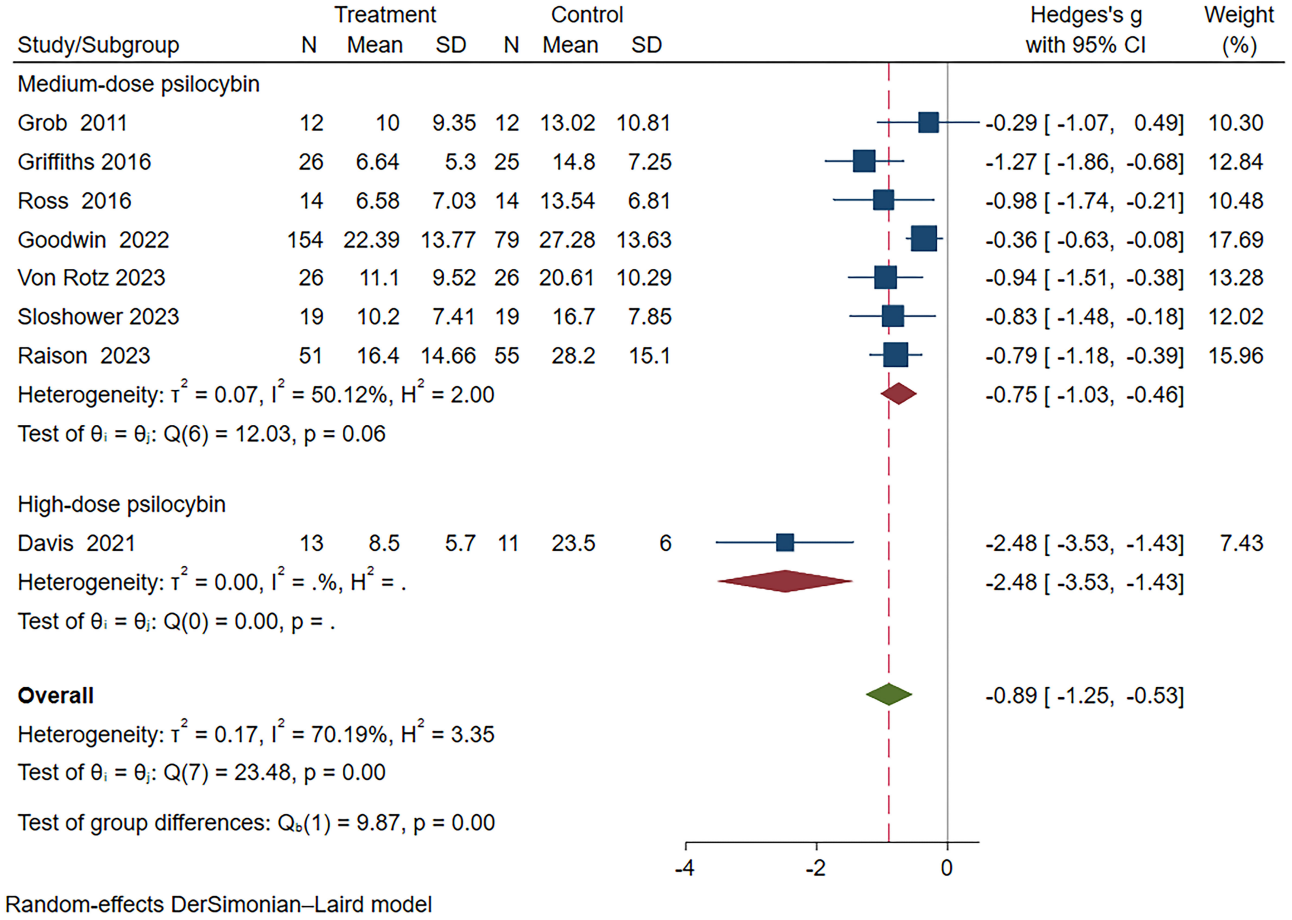
Meta-analysis of psilocybin compared to placebo/low-dose psilocybin for treating primary or secondary depression: subgroup analysis based on dosage.

Subgroup analysis based on different drug dosages revealed that the group receiving medium dosage exhibited a combined effect size of Hedges’ g=-0.75 (95% CI: -1.03 to -0.46, I²=50.12%, P=0.06). In the high dosage group analysis, only Davis et al.’s study (22) was included, demonstrating a Hedges’ g of -2.48 (95%CI: -3.53~-1.43) (**Figure 7**). Unfortunately, none of the studies involving a low dosage group met the inclusion criteria for this subgroup analysis.

### 3.6 Sensitivity analysis

The results of the sensitivity analysis are presented in **Supplementary Figure S1**. A leave-one-out approach was employed to assess the influence of individual studies on the robustness of the meta-analysis findings. The results of our meta-analysis were found to be highly robust.

### 3.7 Response rates

We extracted response rate data from six studies (19, 20, 24–27), and meta-analysis results showed that the response rate of the psilocybin group was better than that of the control group (OR=10.24, 95% CI: 2.51-13.91, I²=66.48%, p<0.01) (see **Supplementary Figure S2.1**). Subgroup analysis of response rates revealed a higher likelihood of response in secondary depression compared to primary depression, with an increasing duration since psilocybin administration correlating positively with the response rate (**Supplementary Figure S2.2**).

### 3.8 Drop-out rates

Eight studies (18–20, 22, 24–27) provided data on dropout rates. One study (18) was excluded from the forest plot because there were no patient withdrawals in either the psilocybin or placebo groups. Among the 7 included studies (19, 20, 22, 24–27), the OR for dropout rate was 0.84 (95% CI: 0.48- 1.49, I²=0%, p=0.55), indicating that there was no significant difference in dropout rates between the psilocybin and placebo groups (**Supplementary Figure S3**).

### 3.9 Adverse events

The data pertaining to adverse events were acquired from a total of nine studies. All eight studies reported reversible adverse effects on the cardiovascular system, and one study (25) did not observe statistically significant differences in vital signs. We collected data on heart rate, systolic blood pressure, and diastolic blood pressure from four studies and performed a chronological meta-analysis of these variables. We observed a statistically significant increase in heart rate in the psilocybin group compared to the control group at 90 minutes after administration, but after 240 minutes, the difference between the two groups disappeared. Changes in systolic and diastolic blood pressure were similar after taking psilocybin. The systolic blood pressure and diastolic blood pressure of the psilocybin group showed significant statistical differences compared with the control group at 60 minutes, until the difference between the two groups disappeared at 360 minutes (**Figures S4-6**).

Six studies reported no serious adverse events, while three studies reported serious adverse events. According to the study (24) conducted by Goodwin et al., 8 out of 154 participants (5.2%) experienced serious adverse events within a timeframe ranging from 2 days to 3 weeks after consuming moderate doses (10-30mg) of psilocybin, including suicidal ideation (2.6%), intentional self-harm (1.9%), and hospitalization (0.6%). No severe adverse events were reported in the low-dose control group throughout this observation period. From 3 to 12 weeks after taking psilocybin, 7 out of 154 subjects (4.5%) in the psilocybin group (10-30mg) had serious adverse events, while only 1 out of 79 subjects (1.3%) in the low-dose psilocybin group (1mg) had such events. In a study (26) conducted by Sloshower et al., it was reported that out of 15 participants, one patient sought hospitalization after a duration of 2 weeks following psilocybin administration due to the lack of improvement in their depressive symptoms. The study (25) conducted by Raison et al. revealed that out of the 50 patients treated with psilocybin, four serious adverse events were reported, including severe migraine (n=1), severe headache (n=1), severe illusion (n=1), and a case involving comorbid severe panic attack and paranoia. Conversely, no serious adverse events were observed in the placebo group. Moreover, in a clinical trial comparing psilocybin with escitalopram for the management of major depressive disorder (MDD) in a cohort of 59 patients (21), it was observed that psilocybin-related adverse effects were generally mild; however, psilocybin exhibited a higher propensity for inducing headaches compared to escitalopram, with incidence rates of 66.7% and 51.7%, respectively.

Eight studies reported symptoms related to the nervous system (e.g., headache, dizziness), mental disorders (e.g., anxiety, depression, sleep disorders), digestive system (e.g., nausea, vomiting), and general symptoms (e.g., fatigue, abnormal physical sensations). Among these studies, six studies provided the necessary data for meta-analysis. The psilocybin group was more likely than the placebo group to develop symptoms related to the digestive system (OR = 13.44, 95%CI: 5.35-33.75, I²=0%, P<0.01), nervous system (OR = 5.67, 95%CI: 1.74 -18.48, I²=72.86%, P<0.01), mental disorder (OR = 4.32, 95%CI: 1.79-10.45, I²=61.01%, P<0.01), and general physical symptoms (OR =2.07, 95%CI: 1.02-4.18, I²=0%, P=0.04) (**Figure S7**).

### 3.10 Publication bias

The presence of publication bias was assessed using a funnel plot and Egger’s regression test. The asymmetrical distribution of data points in the funnel plot suggests the existence of publication bias (**Figure S8**), which was further confirmed by Egger’s regression test(p=0.0165).

## 4 Discussion

### 4.1 Key findings

This meta-analysis, encompassing eight studies involving 524 patients with primary or secondary depression, provides a comprehensive assessment of the efficacy, acceptability, and tolerability of psilocybin in the treatment of depression. Our findings reveal that psilocybin exhibits rapid and enduring antidepressant effects. In comparison to the control group, psilocybin treatment demonstrates significantly stronger antidepressant effects in the primary endpoint, consistent with previous meta-analysis results (29, 30). Ko et al. conducted a meta-analysis of depressive scores between the psilocybin and control groups at different time points, revealing variations in psilocybin efficacy depending on the assessment period. On day 1, the estimated effect sizes (standardized mean difference, SMD) were -1.36 (95% CI: -2.50 to -0.22, p = 0.02), and the maximum effect was observed at 3-5 weeks, with SMD = 3.12 (95% CI: -6.19 to -0.04, p = 0.05). In our study, the pooled effect sizes for studies with assessment periods exceeding 1 month was -0.97, showing significant differences from Ko et al.’s study, likely due to variations in the included studies.

Subgroup analysis results indicate that the efficacy of psilocybin is superior for patients with primary depression compared to those with secondary depression. Higher doses and longer duration of psilocybin treatment are associated with better therapeutic outcomes. In our study, a high dose is defined as exceeding 30mg, suggesting that the optimal dosage for the antidepressant effect of psilocybin may surpass 30 mg. Recent meta-analyses suggest that the optimal dose for alleviating depressive symptoms with psilocybin is approximately 36.08mg/70kg (49), consistent with our study findings. Unfortunately, our study did not include low-dose psilocybin, preventing an assessment of its therapeutic efficacy. However, skepticism has been raised regarding the notion that the microdose effects of psilocybin are solely attributable to placebo-driven expectancy effects, indicating that this perspective may be premature and potentially erroneous (50). Further research is necessary to investigate the efficacy of low-dose psilocybin in treating depression.

Our study revealed that the psilocybin group exhibited a higher incidence of adverse events in the digestive system, nervous system, mental disorders and general physical symptoms compared to the control group. However, there was no significant difference in dropout rates between the two groups, suggesting that psilocybin is a well-tolerated and acceptable intervention.

### 4.2 Review of psilocybin

Psilocybin, a naturally occurring psychedelic found in the Psilocybe genus of mushrooms (51), has been employed for centuries by certain indigenous communities as a means to facilitate spiritual experiences within the framework of sacred rituals (52). In modern society, the use of classic psychedelics is not uncommon among adolescents and young adults, primarily for recreational purposes (53); for example, data from 2001-2004 showed that 21,967 of 130,152 randomly selected people in the United States used psychedelics (54).

As a classic psychedelic, the central function of psilocybin is to act as a 5-HT_2_A receptor agonist (55), which may be the main mechanism by which it can improve depression (56). The therapeutic effects of psilocybin in treating depression can be observed within a day, surpassing the rapidity of nearly all current first-line antidepressants (18, 20, 21, 24, 51, 57), while its efficacy is comparable and not inferior (7, 58, 59). Psilocybin not only induces alterations in sensory perception (52), alleviates psychological distress (60), and augments positive emotions (55), but it is also characterized by low toxicity (61), lacks the potential for dependence or addiction (55), and generally only causes transient and reversible adverse reactions (7, 58, 59).

In conclusion, psilocybin holds promise as a rapid alleviator of negative perceptions and enhancer of mood in individuals suffering from depression.

### 4.3 Drugs comparison

Escitalopram, a novel antidepressant, has gained widespread use owing to its remarkable efficacy and tolerability in the treatment of depression (62, 63). A clinical trial finds no significant difference between psilocybin and escitalopram in relieving depressive symptoms after 6 weeks of treatment and holds the advantage of addressing long-term treatment needs (21). Esketamine, an FDA-approved rapid-acting antidepressant for individuals with TRD (64), has been extensively researched, affirming its effectiveness and safety (65–78). Previous studies have demonstrated that psilocybin not only exhibits comparable efficacy to esketamine but also potentially offers a superior safety profile (70). The results of our meta-analysis similarly demonstrate that psilocybin exhibits rapid antidepressant effects and possesses high acceptability and tolerability, potentially emerging as a novel therapeutic option for depression.

### 4.4 Limitations

Our study presents a comprehensive meta-analysis of the therapeutic efficacy of psilocybin; however, it is important to acknowledge and consider the inherent limitations when interpreting the findings.

Firstly, it should be noted that our study was limited by a relatively small sample size, encompassing only eight major randomized controlled trials. Moreover, a significant level of heterogeneity was observed among the included studies, which can be attributed to variations in populations, sample characteristics, treatment regimens, experimental design, administered doses, or employed outcome measures. We attempted to analyze the number of depressive episodes experienced by participants in the included studies and the means of antidepressant treatment employed during the current episode to assess their impact on the study outcomes and heterogeneity. Unfortunately, we obtained limited information, preventing a comprehensive analysis. Limited sample sizes and substantial between-study heterogeneity may introduce potential bias, diminish the precision of our treatment effect estimates, and restrict the generalizability of our findings (79). Although we conducted subgroup and sensitivity analyses to address these differences, the potential influence of these factors on the overall treatment effect cannot be entirely excluded. Therefore, it is imperative to exercise caution when interpreting the findings of this analysis. Additionally, it is crucial to acknowledge the potential presence of publication bias. Despite our diligent efforts to search for and incorporate unpublished data, the majority of available trial data remains limited or incomplete. We employed analytical techniques such as funnel plots and Egger regression to evaluate the impact of publication bias; regrettably, all results consistently indicated the presence of publication bias. Furthermore, it is important to acknowledge that there was variability in the quality of included studies, with only three studies deemed at low risk of bias. Based on bias risk, we conducted subgroup analyses, revealing that in the low bias risk subgroup, Hedges’ g was -0.64 (95% CI: -1.01 ~ -0.27, I² = 61.78%, p = 0.07) (**Figure S9**). Conversely, in the high bias risk subgroup, Hedges’ g was -1.60 (95% CI: -3.21 ~ 0.01, I² = 85.45%, p = 0.01) (**Figure S9**), indicating that in the high bias risk subgroup, the antidepressant effect of psilocybin was not statistically different from the control group. After excluding two high bias risk studies, we conducted a meta-analysis on studies assessed as low or unclear risk, yielding a combined effect size of Hedges’ g = -0.74 (95% CI: -1.06 ~ -0.42, I² = 57.25%, p < 0.01) (**Figure S10**). Although differences in study quality do influence the results of meta-analysis, the antidepressant effect of psilocybin compared to the control group persisted after excluding high-risk bias studies, albeit with a decrease from the previous Hedges’ g of -0.89. Lastly, we observed that despite the implementation of blinding in the experimental design, participants and researchers often could deduce the allocation based on post-administration reactions. In an effort to enhance blinding and minimize expectancy effects, Sloshower et al. informed participants that they would randomly receive two out of three possible dose conditions: (1) placebo, (2) low-dose psilocybin (0.1mg/kg), and (3) moderate-dose psilocybin (0.3mg/kg). Only placebo and moderate-dose psilocybin were administered during the trial. However, the effectiveness of blinding was less than optimal, as approximately 80% of individuals correctly guessed they had received the moderate dose of psilocybin. In the future, meticulously designed clinical trials are still needed, with particular emphasis on enhancing functional unblinding strategies to mitigate their impact on trial outcomes. Further research is essential to delve into the efficacy of psilocybin.

## 5 Conclusion

In summary, our meta-analysis demonstrates the potential of psilocybin for providing short- and long-term relief from depression, with higher doses of psilocybin exhibiting stronger antidepressant effects. It is important to note that while few studies have reported serious adverse events following psilocybin use, these events cannot be disregarded. Further high-quality randomized controlled trials are necessary to investigate the efficacy of psilocybin in treating depression, particularly in patients with treatment-resistant depression. Additionally, future clinical studies should elucidate the relationship between dosage of psilocybin and both its antidepressant efficacy and adverse effects in order to determine the optimal therapeutic dose for depression treatment.

## Data availability statement

The original contributions presented in the study are included in the article/**Supplementary Material**. Further inquiries can be directed to the corresponding author.

## Author contributions

SF: Conceptualization, Investigation, Methodology, Writing – original draft. XY: Investigation, Methodology, Writing – original draft, Data curation, Software, Visualization. WZ: Supervision, Writing – review & editing.

## References

[1] World Health Organization . Depression. Available online at: https://www.who.int/health-topics/depression#tab=tab_1 (Accessed April 15, 2023).

[2] World Health Organization . International Classification of Diseases 11th Revision . Available online at: https://icd.who.int/en (Accessed April 18, 2023).

[3] Malhi GS Mann JJ . Depression. Lancet (London England). (2018) 392:2299–312. doi: 10.1016/S0140-6736(18)31948-2. 30396512

[4] World Health Organization . WHO Director-General’s opening remarks at the Mental Health at Work panel, World Economic Forum – 18 January 2023 . Available online at: https://www.who.int/director-general/speeches/detail/who-director-general-s-opening-remarks-at-the-mental-health-at-work-panel–world-economic-forum—18-january-2023 (Accessed April 20, 2023).

[5] Li M Yao X Sun L Zhao L Xu W Zhao H . Effects of electroconvulsive therapy on depression and its potential mechanism. Front Psychol (2020) 11:80. doi: 10.3389/fpsyg.2020.00080. 32153449 PMC7044268

[6] Sabella D . Antidepressant medications. Am J Nursing (2018) 118:52–9. doi: 10.1097/01.NAJ.0000544978.56301.f6. 30138204

[7] Wang SM Han C Bahk WM Lee SJ Patkar AA Masand PS . Addressing the side effects of contemporary antidepressant drugs: A comprehensive review. Chonnam Med J (2018) 54:101–12. doi: 10.4068/cmj.2018.54.2.101. PMC5972123 29854675

[8] Hung CI . Factors predicting adherence to antidepressant treatment. Curr Opin Psychiatry (2014) 27:344–9. doi: 10.1097/YCO.0000000000000086. 25033275

[9] Howland RH . Sequenced treatment alternatives to relieve depression (STAR*D). Part 2: study outcomes. J psychosocial Nurs Ment Health Serv (2008) 46:21–4. doi: 10.3928/02793695-20081001-05. 18935932

[10] Rush AJ Trivedi MH Wisniewski SR Nierenberg AA Stewart JW Warden D . Acute and longer-term outcomes in depressed outpatients requiring one or several treatment steps: a STAR*D report. Am J Psychiatry (2006) 163:1905–17. doi: 10.1176/ajp.2006.163.11.1905. 17074942

[11] Weiner RD Reti IM . Key updates in the clinical application of electroconvulsive therapy. Int Rev Psychiatry (Abingdon England) (2017) 29:54–62. doi: 10.1080/09540261.2017.1309362. 28406327

[12] Prapotnik M Pycha R Nemes C König P Hausmann A Conca A . [Adverse cognitive effects and ECT]. Wiener medizinische Wochenschrift (1946). (2006) 156:200–8. doi: 10.1007/s10354-005-0237-6 16823537

[13] Kelmendi B Kaye AP Pittenger C Kwan AC . Psychedelics. Curr biology: CB. (2022) 32:R63–r7. doi: 10.1016/j.cub.2021.12.009. PMC8830367 35077687

[14] Madsen MK Fisher PM Burmester D Dyssegaard A Stenbæk DS Kristiansen S . Psychedelic effects of psilocybin correlate with serotonin 2A receptor occupancy and plasma psilocin levels. Neuropsychopharmacology (2019) 44:1328–34. doi: 10.1038/s41386-019-0324-9. PMC6785028 30685771

[15] Griffiths RR Richards WA McCann U Jesse R . Psilocybin can occasion mystical-type experiences having substantial and sustained personal meaning and spiritual significance. Psychopharmacology (2006) 187:268–83; discussion 84-92. doi: 10.1007/s00213-006-0457-5. 16826400

[16] Carbonaro TM Bradstreet MP Barrett FS MacLean KA Jesse R Johnson MW . Survey study of challenging experiences after ingesting psilocybin mushrooms: Acute and enduring positive and negative consequences. J Psychopharmacol (Oxford England). (2016) 30:1268–78. doi: 10.1177/0269881116662634. PMC5551678 27578767

[17] Hartman S . Psilocybin Could Be Legal for Therapy by 2021 (2018). Available online at: https://www.rollingstone.com/culture/culture-news/psilocybin-legal-therapy-mdma-753946/ (Accessed May 1, 2023).

[18] Grob CS Danforth AL Chopra GS Hagerty M McKay CR Halberstadt AL . Pilot study of psilocybin treatment for anxiety in patients with advanced-stage cancer. Arch Gen Psychiatry (2011) 68:71–8. doi: 10.1001/archgenpsychiatry.2010.116. 20819978

[19] Griffiths RR Johnson MW Carducci MA Umbricht A Richards WA Richards BD . Psilocybin produces substantial and sustained decreases in depression and anxiety in patients with life-threatening cancer: A randomized double-blind trial. J Psychopharmacol (Oxford England) (2016) 30:1181–97. doi: 10.1177/0269881116675513. PMC5367557 27909165

[20] Ross S Bossis A Guss J Agin-Liebes G Malone T Cohen B . Rapid and sustained symptom reduction following psilocybin treatment for anxiety and depression in patients with life-threatening cancer: a randomized controlled trial. J Psychopharmacol (Oxford England) (2016) 30:1165–80. doi: 10.1177/0269881116675512. PMC5367551 27909164

[21] Carhart-Harris R Giribaldi B Watts R Baker-Jones M Murphy-Beiner A Murphy R . Trial of psilocybin versus escitalopram for depression. New Engl J Med (2021) 384:1402–11. doi: 10.1056/NEJMoa2032994. 33852780

[22] Davis AK Barrett FS May DG Cosimano MP Sepeda ND Johnson MW . Effects of psilocybin-assisted therapy on major depressive disorder: A randomized clinical trial. JAMA Psychiatry (2021) 78:481–9. doi: 10.1001/jamapsychiatry.2020.3285. PMC7643046 33146667

[23] Gukasyan N Davis AK Barrett FS Cosimano MP Sepeda ND Johnson MW . Efficacy and safety of psilocybin-assisted treatment for major depressive disorder: Prospective 12-month follow-up. J Psychopharmacol (Oxford England) (2022) 36:151–8. doi: 10.1177/02698811211073759. PMC8864328 35166158

[24] Goodwin GM Aaronson ST Alvarez O Arden PC Baker A Bennett JC . Single-dose psilocybin for a treatment-resistant episode of major depression. New Engl J Med (2022) 387:1637–48. doi: 10.1056/NEJMoa2206443 36322843

[25] Raison CL Sanacora G Woolley J Heinzerling K Dunlop BW Brown RT . Single-dose psilocybin treatment for major depressive disorder: A randomized clinical trial. Jama (2023) 330:843–53. doi: 10.1001/jama.2023.14530. PMC10472268 37651119

[26] Sloshower J Skosnik PD Safi-Aghdam H Pathania S Syed S Pittman B . Psilocybin-assisted therapy for major depressive disorder: An exploratory placebo-controlled, fixed-order trial. J Psychopharmacol (Oxford England) (2023) 37:698–706. doi: 10.1177/02698811231154852. 36938991

[27] von Rotz R Schindowski EM Jungwirth J Schuldt A Rieser NM Zahoranszky K . Single-dose psilocybin-assisted therapy in major depressive disorder: A placebo-controlled, double-blind, randomised clinical trial. EClinicalMedicine (2023) 56:101809. doi: 10.1016/j.eclinm.2022.101809. 36636296 PMC9830149

[28] Goldberg SB Pace BT Nicholas CR Raison CL Hutson PR . The experimental effects of psilocybin on symptoms of anxiety and depression: A meta-analysis. Psychiatry Res (2020) 284:112749. doi: 10.1016/j.psychres.2020.112749. 31931272

[29] Yu CL Liang CS Yang FC Tu YK Hsu CW Carvalho AF . Trajectory of antidepressant effects after single- or two-dose administration of psilocybin: A systematic review and multivariate meta-analysis. J Clin Med (2022) 11. doi: 10.3390/jcm11040938. PMC8879743 35207210

[30] Ko K Kopra EI Cleare AJ Rucker JJ . Psychedelic therapy for depressive symptoms: A systematic review and meta-analysis. J Affect Disord (2023) 322:194–204. doi: 10.1016/j.jad.2022.09.168. 36209780

[31] Romeo B Karila L Martelli C Benyamina A . Efficacy of psychedelic treatments on depressive symptoms: A meta-analysis. J Psychopharmacol (Oxford England). (2020) 34:1079–85. doi: 10.1177/0269881120919957. 32448048

[32] Leger RF Unterwald EM . Assessing the effects of methodological differences on outcomes in the use of psychedelics in the treatment of anxiety and depressive disorders: A systematic review and meta-analysis. J Psychopharmacol (Oxford England). (2022) 36:20–30. doi: 10.1177/02698811211044688. 34519567

[33] Haikazian S Chen-Li DCJ Johnson DE Fancy F Levinta A Husain MI . Psilocybin-assisted therapy for depression: A systematic review and meta-analysis. Psychiatry Res (2023) 329:115531. doi: 10.1016/j.psychres.2023.115531. 37844352

[34] Higgins JPT TJ Chandler J Cumpston M Li T Page MJ Welch VA . Cochrane Handbook for Systematic Reviews of Interventions. 2nd Edition. Chichester (UK: John Wiley & Sons (2019).

[35] Page MJ McKenzie JE Bossuyt PM Boutron I Hoffmann TC Mulrow CD . The PRISMA 2020 statement: an updated guideline for reporting systematic reviews. BMJ (Clinical Res ed). (2021) 372:n71. doi: 10.1136/bmj.n71. PMC8005924 33782057

[36] Montgomery SA Asberg M . A new depression scale designed to be sensitive to change. Br J psychiatry: J Ment science (1979) 134:382–9. doi: 10.1192/bjp.134.4.382. 444788

[37] Hamilton M . A rating scale for depression. J Neurol Neurosurgery Psychiatry (1960) 23:56–62. doi: 10.1136/jnnp.23.1.56. PMC495331 14399272

[38] Beck AT Steer RA Ball R Ranieri W . Comparison of Beck Depression Inventories -IA and -II in psychiatric outpatients. J Pers Assessment (1996) 67:588–97. doi: 10.1207/s15327752jpa6703_13. 8991972

[39] Rush AJ Trivedi MH Ibrahim HM Carmody TJ Arnow B Klein DN . The 16-Item Quick Inventory of Depressive Symptomatology (QIDS), clinician rating (QIDS-C), and self-report (QIDS-SR): a psychometric evaluation in patients with chronic major depression. Biol Psychiatry (2003) 54:573–83. doi: 10.1016/S0006-3223(02)01866-8. 12946886

[40] Williams JBW Kobak KA Bech P Engelhardt N Evans K Lipsitz J . The GRID-HAMD: standardization of the hamilton depression rating scale. Int Clin Psychopharmacol (2008) 23:120–9. doi: 10.1097/YIC.0b013e3282f948f5. 18408526

[41] Furukawa TA Salanti G Atkinson LZ Leucht S Ruhe HG Turner EH . Comparative efficacy and acceptability of first-generation and second-generation antidepressants in the acute treatment of major depression: protocol for a network meta-analysis. BMJ Open (2016) 6:e010919. doi: 10.1136/bmjopen-2015-010919. PMC4947714 27401359

[42] Parmar MK Torri V Stewart L . Extracting summary statistics to perform meta-analyses of the published literature for survival endpoints. Stat Med (1998) 17:2815–34. doi: 10.1002/(ISSN)1097-0258. 9921604

[43] Wojtyniak JG Britz H Selzer D Schwab M Lehr T . Data digitizing: accurate and precise data extraction for quantitative systems pharmacology and physiologically-based pharmacokinetic modeling. CPT Pharmacometrics Syst Pharmacol (2020) 9:322–31. doi: 10.1002/psp4.12511. PMC7306621 32543786

[44] Sterne JAC Savović J Page MJ Elbers RG Blencowe NS Boutron I . RoB 2: a revised tool for assessing risk of bias in randomised trials. BMJ (Clinical Res ed). (2019) 366:l4898. doi: 10.1136/bmj.l4898. 31462531

[45] Olkin I Hedges LV . Statistical Methods for Meta-Analysis (New York, USA: Academic Press) (1985).

[46] Higgins JP Thompson SG Deeks JJ Altman DG . Measuring inconsistency in meta-analyses. BMJ (Clinical Res ed). (2003) 327:557–60. doi: 10.1136/bmj.327.7414.557. PMC192859 12958120

[47] Borenstein M Hedges LV Higgins JP Rothstein HR . A basic introduction to fixed-effect and random-effects models for meta-analysis. Res synthesis Methods (2010) 1:97–111. doi: 10.1002/jrsm.12. 26061376

[48] Egger M Davey Smith G Schneider M Minder C . Bias in meta-analysis detected by a simple, graphical test. BMJ (Clinical Res ed) (1997) 315:629–34. doi: 10.1136/bmj.315.7109.629. PMC2127453 9310563

[49] Perez N Langlest F Mallet L De Pieri M Sentissi O Thorens G . Psilocybin-assisted therapy for depression: A systematic review and dose-response meta-analysis of human studies. Eur Neuropsychopharmacol (2023) 76:61–76. doi: 10.1016/j.euroneuro.2023.07.011. 37557019

[50] Polito V Liknaitzky P . The emerging science of microdosing: A systematic review of research on low dose psychedelics (1955-2021) and recommendations for the field. Neurosci Biobehav Rev (2022) 139:104706. doi: 10.1016/j.neubiorev.2022.104706. 35609684

[51] Ling S Ceban F Lui LMW Lee Y Teopiz KM Rodrigues NB . Molecular mechanisms of psilocybin and implications for the treatment of depression. CNS Drugs (2022) 36:17–30. doi: 10.1007/s40263-021-00877-y. 34791625

[52] Reiff CM Richman EE Nemeroff CB Carpenter LL Widge AS Rodriguez CI . Psychedelics and psychedelic-assisted psychotherapy. Am J Psychiatry (2020) 177:391–410. doi: 10.1176/appi.ajp.2019.19010035. 32098487

[53] Johnson MW Hendricks PS Barrett FS Griffiths RR . Classic psychedelics: An integrative review of epidemiology, therapeutics, mystical experience, and brain network function. Pharmacol Ther (2019) 197:83–102. doi: 10.1016/j.pharmthera.2018.11.010. 30521880

[54] Krebs TS Johansen P . Psychedelics and mental health: a population study. PloS One (2013) 8:e63972. doi: 10.1371/journal.pone.0063972. 23976938 PMC3747247

[55] Nichols DE . Psychedelics. Pharmacol Rev (2016) 68:264–355. doi: 10.1124/pr.115.011478. 26841800 PMC4813425

[56] Albert PR Benkelfat C Descarries L . The neurobiology of depression–revisiting the serotonin hypothesis. I. Cellular and molecular mechanisms. Philos Trans R Soc London Ser B Biol Sci (2012) 367:2378–81. doi: 10.1098/rstb.2012.0190. PMC3405681 22826338

[57] Montgomery SA . Rapid onset of action of venlafaxine. Int Clin Psychopharmacol (1995) 10 Suppl 2:21–7. doi: 10.1097/00004850-199503002-00005. 7622814

[58] Mithoefer MC Grob CS Brewerton TD . Novel psychopharmacological therapies for psychiatric disorders: psilocybin and MDMA. Lancet Psychiatry (2016) 3:481–8. doi: 10.1016/S2215-0366(15)00576-3. 27067625

[59] Todorović Vukotić N Đorđević J Pejić S Đorđević N Pajović SB . Antidepressants- and antipsychotics-induced hepatotoxicity. Arch toxicol (2021) 95:767–89. doi: 10.1007/s00204-020-02963-4. PMC7781826 33398419

[60] Hendricks PS Johnson MW Griffiths RR . Psilocybin, psychological distress, and suicidality. J Psychopharmacol (Oxford England). (2015) 29:1041–3. doi: 10.1177/0269881115598338. PMC4721603 26395582

[61] Passie T Seifert J Schneider U Emrich HM . The pharmacology of psilocybin. Addict Biol (2002) 7:357–64. doi: 10.1080/1355621021000005937. 14578010

[62] Sanchez C Reines EH Montgomery SA . A comparative review of escitalopram, paroxetine, and sertraline: Are they all alike? Int Clin Psychopharmacol (2014) 29:185–96. doi: 10.1097/YIC.0000000000000023. PMC4047306 24424469

[63] Hirschfeld RM Vornik LA . Newer antidepressants: review of efficacy and safety of escitalopram and duloxetine. J Clin Psychiatry (2004) 65 Suppl 4:46–52. 15046541

[64] Food and Drug Administration . FDA approves new nasal spray medication for treatment-resistant depression; available only at a certified doctor’s office or clinic (2019). Available online at: https://www.fda.gov/news-events/press-announcements/fda-approves-new-nasal-spray-medication-treatment-resistant-depression-available-only-certified (Accessed April 28, 2023).

[65] Popova V Daly EJ Trivedi M Cooper K Lane R Lim P . Efficacy and safety of flexibly dosed esketamine nasal spray combined with a newly initiated oral antidepressant in treatment-resistant depression: A randomized double-blind active-controlled study. Am J Psychiatry (2019) 176:428–38. doi: 10.1176/appi.ajp.2019.19020172. 31109201

[66] Nikayin S Murphy E Krystal JH Wilkinson ST . Long-term safety of ketamine and esketamine in treatment of depression. Expert Opin Drug safety (2022) 21:777–87. doi: 10.1080/14740338.2022.2066651. 35416105

[67] Fedgchin M Trivedi M Daly EJ Melkote R Lane R Lim P . Efficacy and safety of fixed-dose esketamine nasal spray combined with a new oral antidepressant in treatment-resistant depression: results of a randomized, double-blind, active-controlled study (TRANSFORM-1). Int J Neuropsychopharmacol (2019) 22:616–30. doi: 10.1093/ijnp/pyz039. PMC6822141 31290965

[68] Vázquez GH Bahji A Undurraga J Tondo L Baldessarini RJ . Efficacy and Tolerability of Combination Treatments for Major Depression: Antidepressants plus Second-Generation Antipsychotics vs. Esketamine vs. Lithium. J Psychopharmacol (Oxford England). (2021) 35:890–900. doi: 10.1177/02698811211013579. PMC8358538 34238049

[69] Daly EJ Singh JB Fedgchin M Cooper K Lim P Shelton RC . Efficacy and safety of intranasal esketamine adjunctive to oral antidepressant therapy in treatment-resistant depression: A randomized clinical trial. JAMA Psychiatry (2018) 75:139–48. doi: 10.1001/jamapsychiatry.2017.3739. PMC5838571 29282469

[70] Psiuk D Nowak EM Dycha N Lopuszanska U Kurzepa J Samardakiewicz M . Esketamine and psilocybin-the comparison of two mind-altering agents in depression treatment: systematic review. Int J Mol Sci (2022) 23. doi: 10.3390/ijms231911450. PMC9570062 36232748

[71] Sapkota A Khurshid H Qureshi IA Jahan N Went TR Sultan W . Efficacy and safety of intranasal esketamine in treatment-resistant depression in adults: A systematic review. Cureus (2021) 13:e17352. doi: 10.7759/cureus.17352. 34447651 PMC8381465

[72] Swainson J Thomas RK Archer S Chrenek C MacKay MA Baker G . Esketamine for treatment resistant depression. Expert Rev neurotherapeutics (2019) 19:899–911. doi: 10.1080/14737175.2019.1640604. 31282772

[73] Bahji A Zarate CA Vazquez GH . Efficacy and safety of racemic ketamine and esketamine for depression: a systematic review and meta-analysis. Expert Opin Drug safetys (2022) 21:853–66. doi: 10.1080/14740338.2022.2047928. PMC9949988 35231204

[74] Xiong J Lipsitz O Chen-Li D Rosenblat JD Rodrigues NB Carvalho I . The acute antisuicidal effects of single-dose intravenous ketamine and intranasal esketamine in individuals with major depression and bipolar disorders: A systematic review and meta-analysis. J Psychiatr Res (2021) 134:57–68. doi: 10.1016/j.jpsychires.2020.12.038. 33360864

[75] Canuso CM Singh JB Fedgchin M Alphs L Lane R Lim P . Efficacy and safety of intranasal esketamine for the rapid reduction of symptoms of depression and suicidality in patients at imminent risk for suicide: results of a double-blind, randomized, placebo-controlled study. Am J Psychiatry (2018) 175:620–30. doi: 10.1176/appi.ajp.2018.17060720. 29656663

[76] Daly EJ Turkoz I Salvadore G Fedgchin M Ionescu DF Starr HL . The effect of esketamine in patients with treatment-resistant depression with and without comorbid anxiety symptoms or disorder. Depression anxiety (2021) 38:1120–30. doi: 10.1002/da.23193. PMC9291524 34293233

[77] Correia-Melo FS Leal GC Vieira F Jesus-Nunes AP Mello RP Magnavita G . Efficacy and safety of adjunctive therapy using esketamine or racemic ketamine for adult treatment-resistant depression: A randomized, double-blind, non-inferiority study. J Affect Disord (2020) 264:527–34. doi: 10.1016/j.jad.2019.11.086. 31786030

[78] McIntyre RS Rosenblat JD Nemeroff CB Sanacora G Murrough JW Berk M . Synthesizing the evidence for ketamine and esketamine in treatment-resistant depression: an international expert opinion on the available evidence and implementation. Am J Psychiatry (2021) 178:383–99. doi: 10.1176/appi.ajp.2020.20081251. PMC9635017 33726522

[79] Button KS Ioannidis JP Mokrysz C Nosek BA Flint J Robinson ES . Power failure: why small sample size undermines the reliability of neuroscience. Nat Rev Neurosci (2013) 14:365–76. doi: 10.1038/nrn3475. 23571845

