# Supplementary material for: Efficacy and acceptability of psilocybin for primary or secondary depression: A systematic review and meta-analysis of randomized controlled trials
Source: Front Psychiatry. 2024 Feb 15;15:1359088. doi: 10.3389/fpsyt.2024.1359088 (PMC10902050; doi:10.3389/fpsyt.2024.1359088)
Supplement: Supplementary file 1 [file DataSheet_1.docx]

Supplementary Material


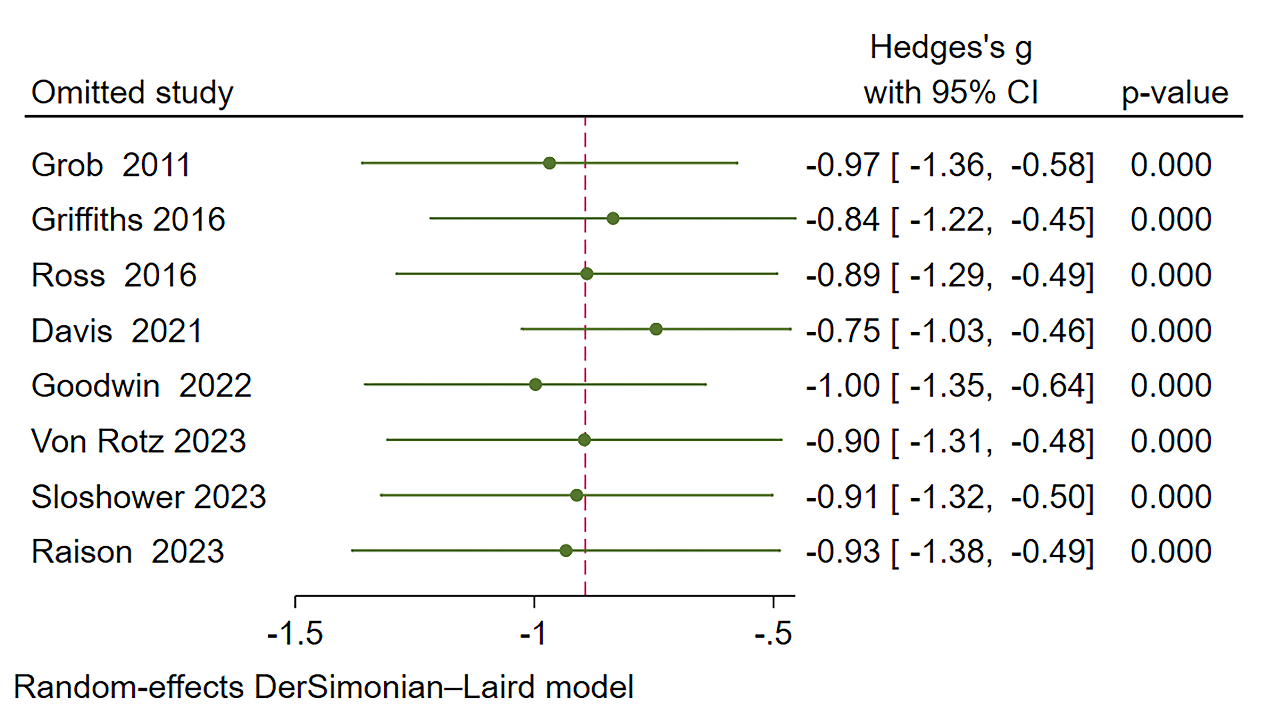


Figure S1: Leave-one-out sensitivity analysis for the meta-analysis of psilocybin vs placebo/low-dose psilocybin in patients with primary or secondary depression. CI, Confidence interval.


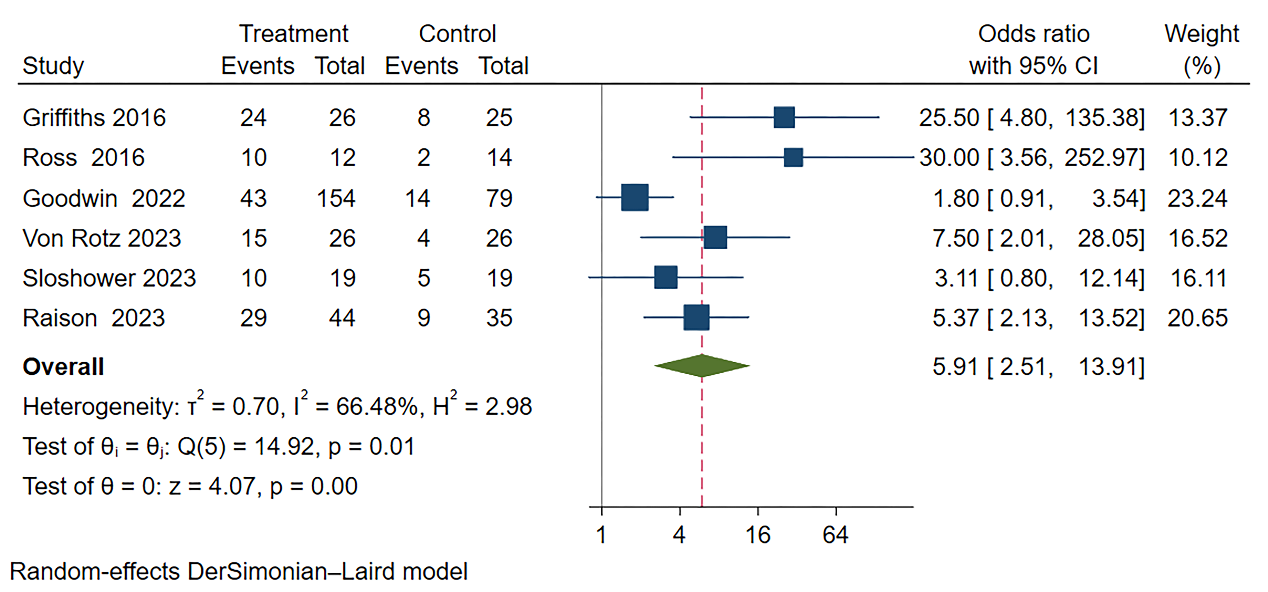


Figure S2.1: Meta-analysis of psilocybin versus placebo/low-dose psilocybin for patients with primary or secondary depression: response rates. CI, Confidence interval.


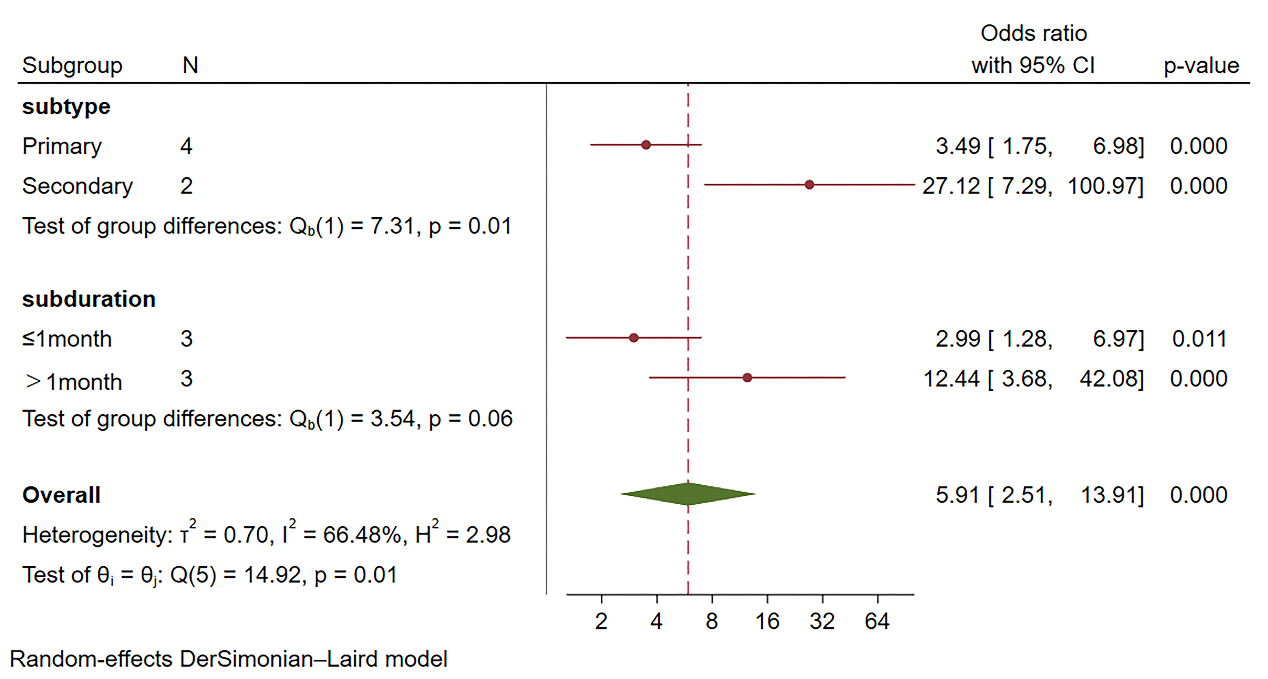


Fig.S2.2: The results of subgroup analyses of psilocybin versus placebo/low-dose psilocybin for patients with primary or secondary depression: response rates. CI: Confidence interval. N: number of included studies.


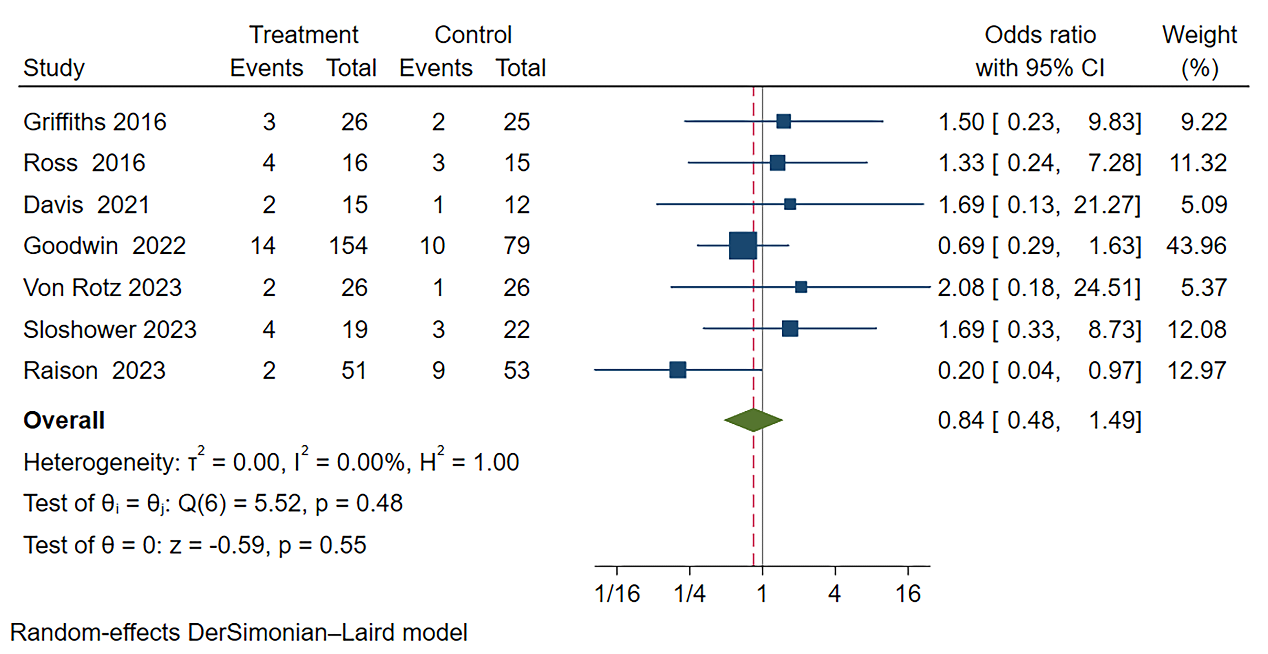


Figure S3: Meta-analysis of psilocybin versus placebo/low-dose psilocybin for patients with primary or secondary depression: drop-out rates. CI: Confidence interval.


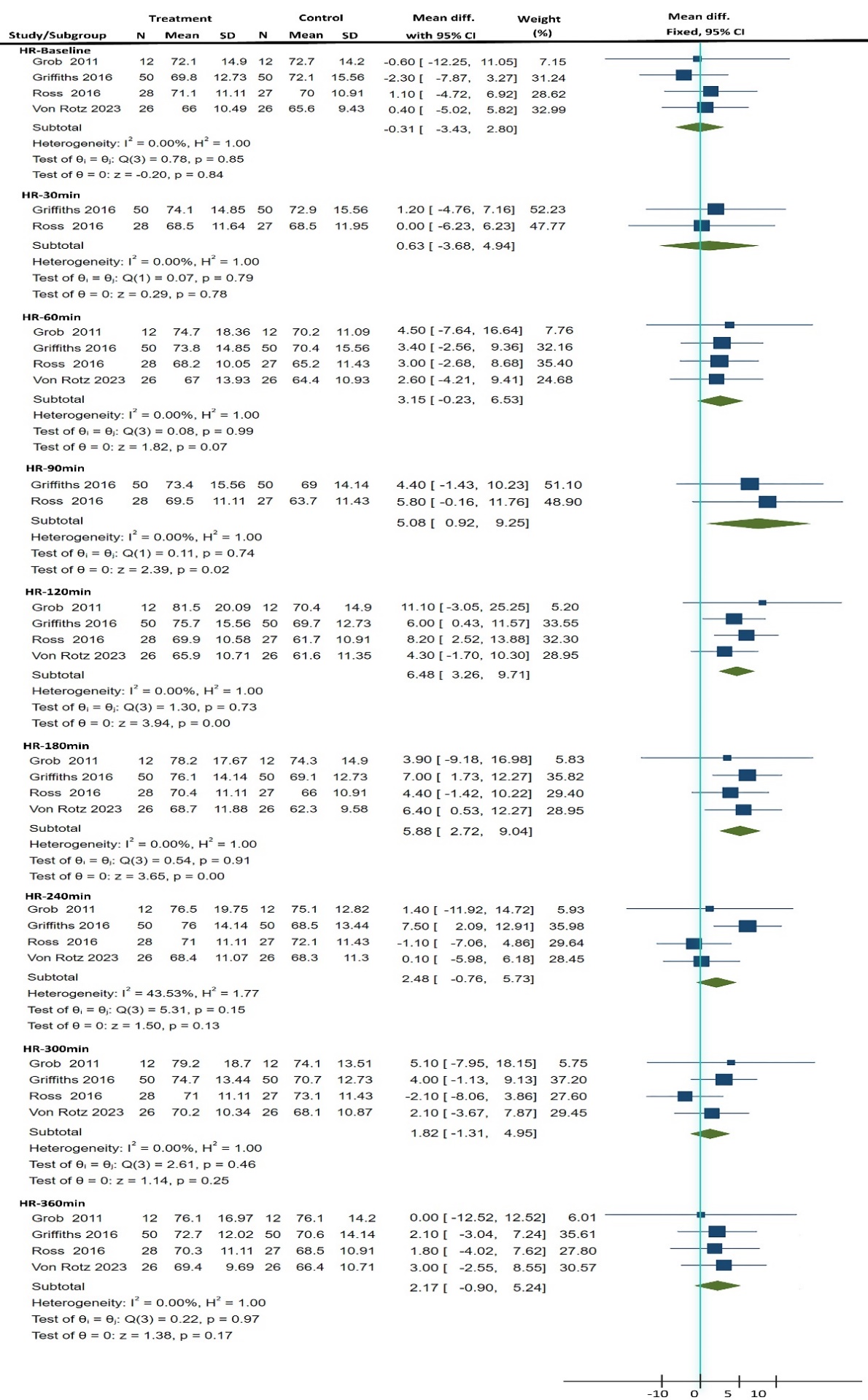


Figure S4: Meta-analysis of psilocybin versus placebo/low-dose psilocybin for patients with primary or secondary depression: heart rates. HR: Heart rates. CI: Confidence interval.


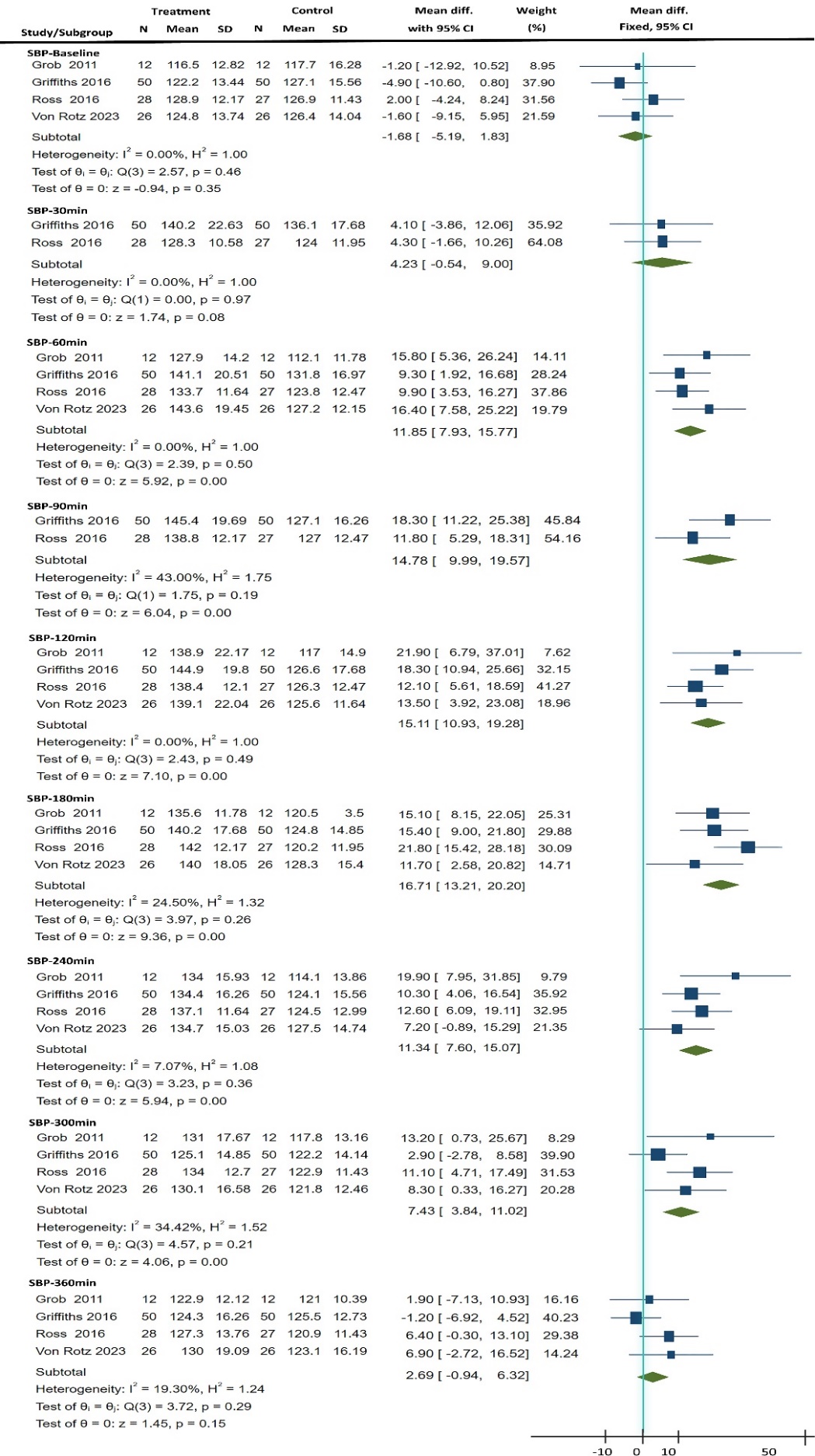


Figure S5: Meta-analysis of psilocybin versus placebo/low-dose psilocybin for patients with primary or secondary depression: SBP. SBP: Systolic blood pressure. CI: Confidence interval.


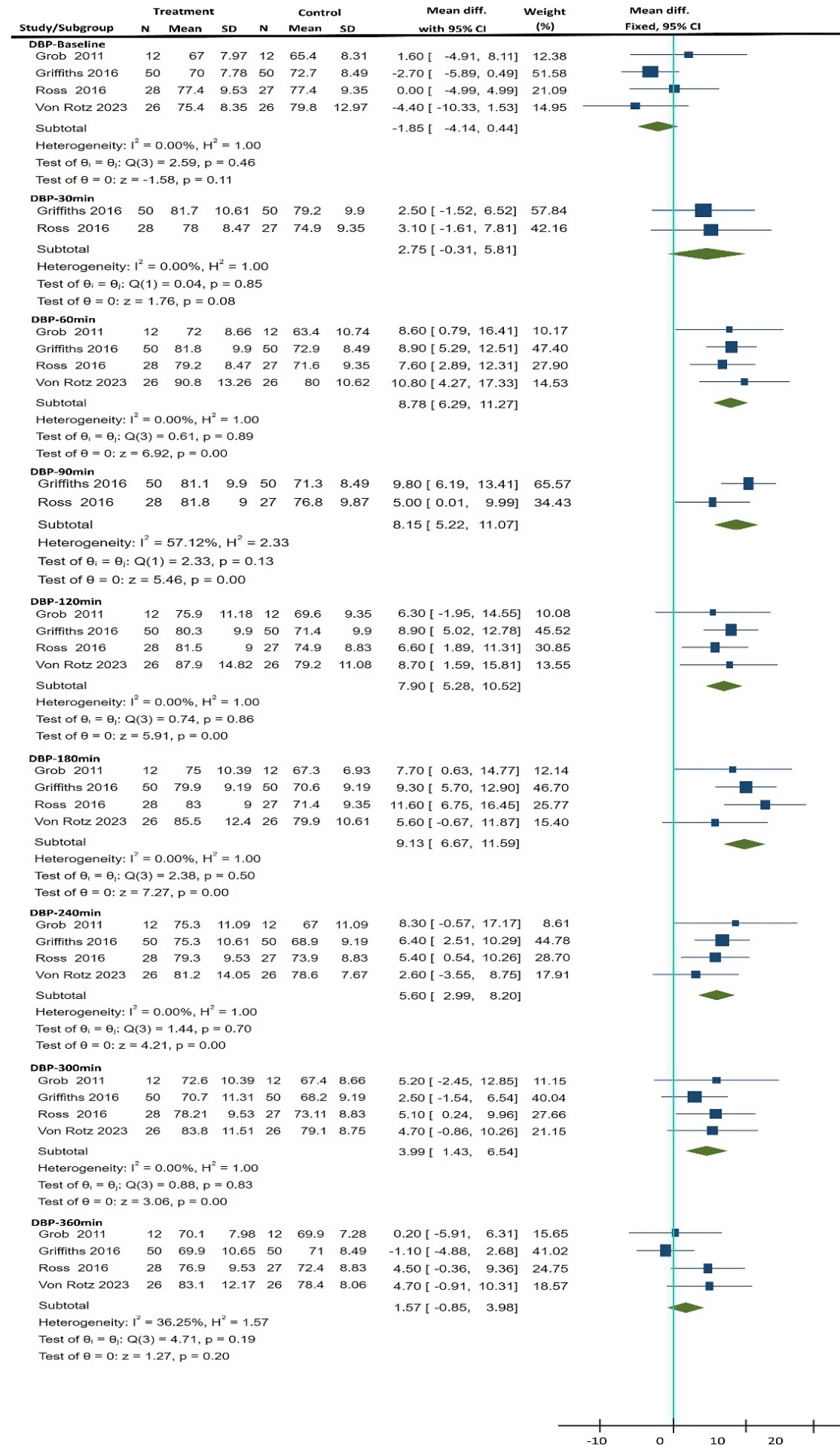


Figure S6: Meta-analysis of psilocybin versus placebo/low-dose psilocybin for patients with primary or secondary depression: DBP. DBP: Diastolic blood pressure. CI: Confidence interval.


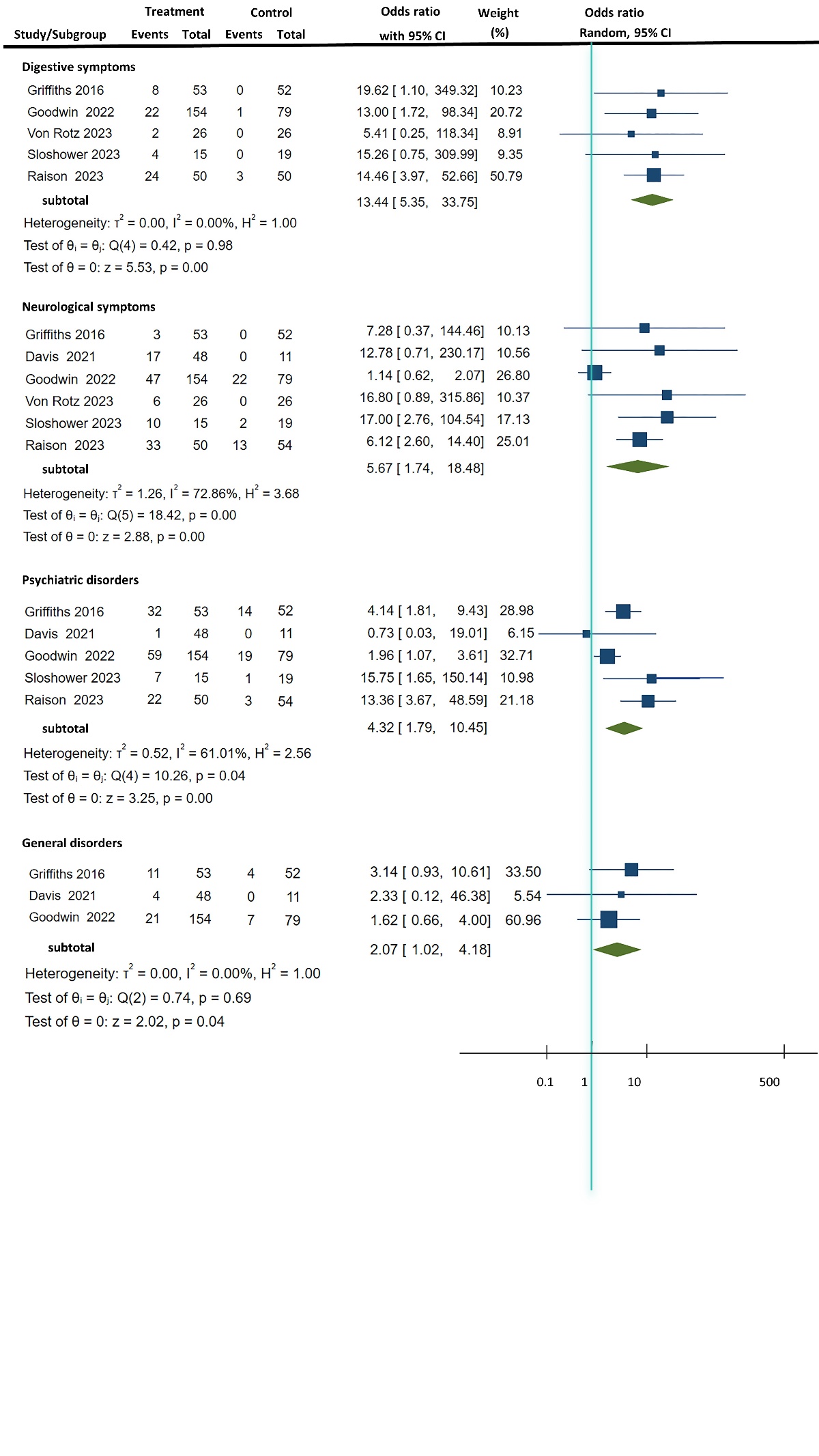


Figure S7: Meta-analysis of psilocybin versus placebo/low-dose psilocybin for patients with primary or secondary depression: adverse events. CI: Confidence interval.


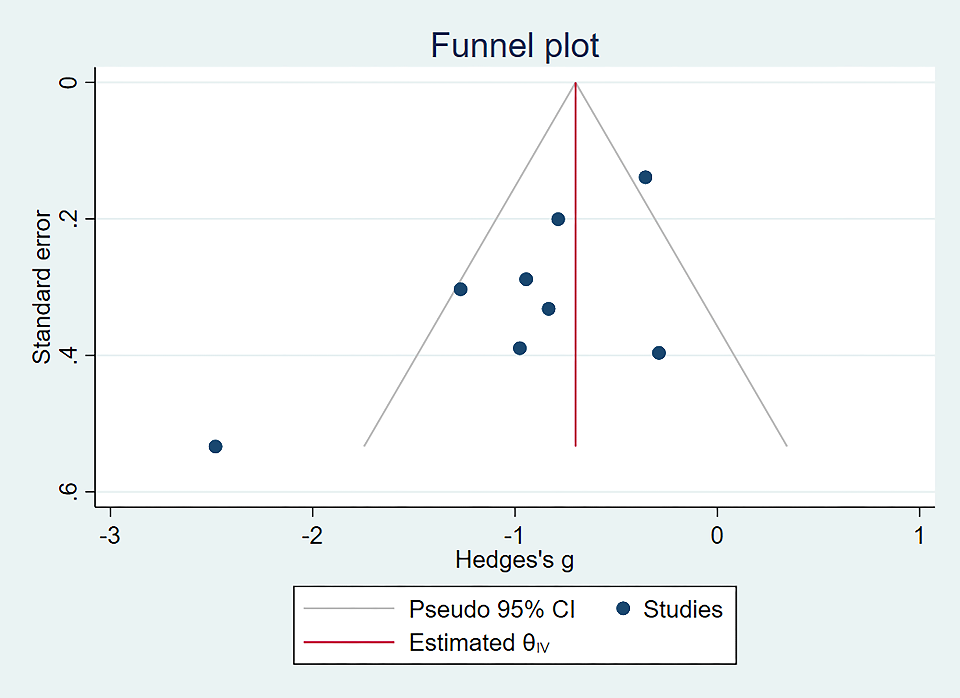


Figure S8: Funnel plot of psilocybin versus placebo/low-dose psilocybin meta-analysis. where the standard error (SE) of each Hedges' g is plotted.


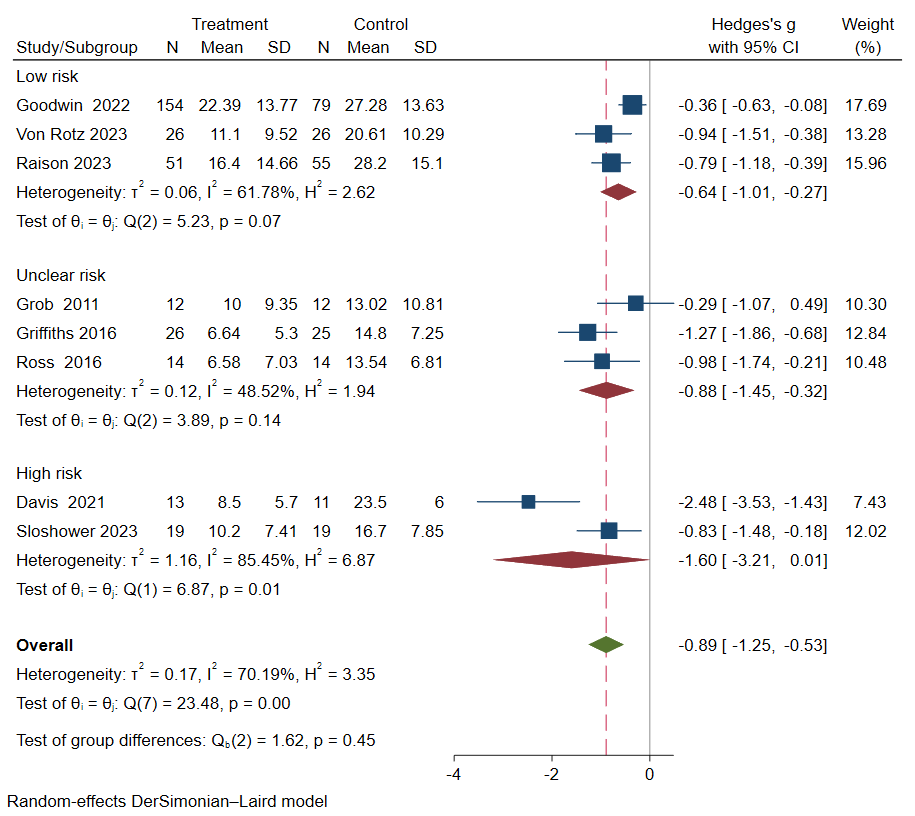


Figure S9: Hedges' g for all included studies grouped by risk of bias.


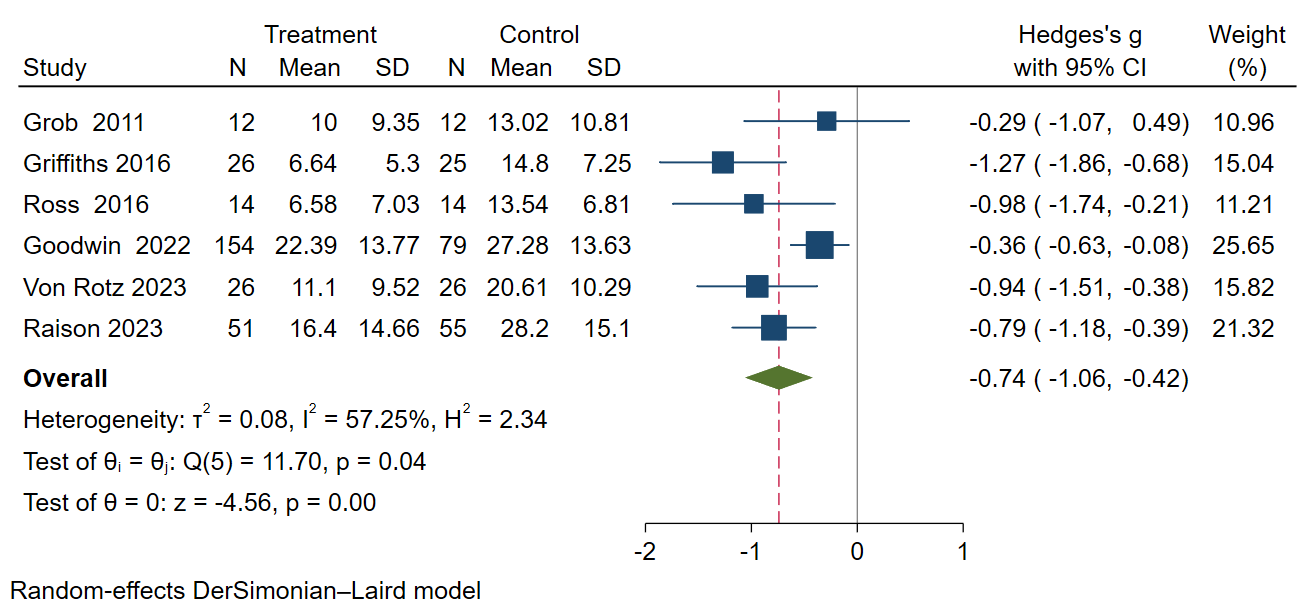


Figure S10: Hedges' g for low risk and unclear risk studies.
